# DriverNet: A Clinical and MRI-Based Framework for Noninvasive Pre-Treatment Molecular Triage in NSCLC Brain Metastases

**DOI:** 10.3390/diagnostics16131988

**Published:** 2026-06-26

**Authors:** Hongliang Mao, Xinyu Wang, Lijuan Wan, Fengchun Mu, Chen Yang, Jinghai Wan, Ming Shan, Hongmei Zhang, Ming Yang

**Affiliations:** 1 Department of Neurosurgery, National Cancer Center/National Clinical Research Center for Cancer/Cancer Hospital, Chinese Academy of Medical Sciences and Peking Union Medical College, No. 17 Panjiayuan Nanli, Chaoyang District, Beijing 100021, China; 2 Department of Radiology, National Cancer Center/National Clinical Research Center for Cancer/Cancer Hospital, Chinese Academy of Medical Sciences and Peking Union Medical College, No. 17 Panjiayuan Nanli, Chaoyang District, Beijing 100021, China; 3 Department of Neurosurgery, The First Affiliated Hospital of Anhui Medical University, Jixi Road 218, Hefei 230022, China

**Keywords:** non-small-cell lung cancer brain metastases, EGFR/ALK status, multimodal MRI, molecular triage, deep learning

## Abstract

**Background/Objectives:** Brain metastases (BMs) are a major cause of morbidity and mortality in non-small-cell lung cancer (NSCLC). In this setting, EGFR-mutant and ALK-rearranged tumors represent clinically actionable, CNS-relevant oncogenic subgroups for which matched TKIs are essential to management, yet lesion-level molecular profiling is not always feasible or immediately available. We aimed to develop and externally validate DriverNet, a clinical and MRI-based framework for noninvasive pre-treatment molecular triage of EGFR/ALK status in NSCLC-BM. **Methods:** In this multicenter study, we analyzed pretreatment clinical, T1CE and T2-FLAIR MRI data to develop unimodal radiomics, 2D/2.5D deep learning (DL), and multimodal fusion models. The final model used ImageNet-pretrained CNNs for feature extraction and a Transformer-based architecture for fusion. The primary cohort was split strictly at the patient level before slice extraction and model development, and two independent external cohorts were used for testing. Clinical-only, imaging-only, and clinical-imaging models were compared using discrimination, calibration, Brier score, and decision-curve analyses. Model interpretability and exploratory prognostic stratification were also assessed. **Results:** A total of 374 patients from three centers were included. Center 1 comprised 224 patients (Chinese) and was divided into training (*n* = 179, EGFR/ALK+ 55.3%) and internal validation (*n* = 45, EGFR/ALK+ 57.8%) sets. External cohorts included 54 patients from Center 2 (Chinese, test 1, EGFR/ALK+ 42.6%) and 96 from Center 3 (Western, test 2, EGFR/ALK+ 12.5%). Among all evaluated models, DriverNet achieved the best overall performance, with AUCs of 0.967, 0.947, 0.962, and 0.952 in the training, internal validation, and two external cohorts, respectively, outperforming the clinical-only and imaging-only models. Model-derived labels were also associated with overall survival in exploratory analyses. **Conclusions:** DriverNet is a clinical and MRI-based framework for noninvasive pre-treatment molecular triage in NSCLC-BM. It may provide complementary information for future molecular triage studies when lesion-level profiling is unavailable or delayed. Prospective validation in larger and more molecularly balanced cohorts remains necessary before any clinical implementation can be considered.

## 1. Introduction

Brain metastasis (BM) is a common and devastating complication of non-small-cell lung cancer (NSCLC) and has historically been associated with a median survival of about 7 months in unselected cohorts [1,2,3]. NSCLC accounts for 80–85% of lung cancers [1], and BM is detected in 7–10% of patients at diagnosis. Across the disease course, the cumulative incidence typically reaches 20–40%, reflecting both the propensity of NSCLC to seed the brain and improved extracranial control that reveals intracranial progression [4].

Management of NSCLC-BM depends on intracranial disease burden, extracranial disease status, symptoms, lesion location, and molecular profile [5,6,7]. In precision oncology, epidermal growth factor receptor (EGFR) mutations and anaplastic lymphoma kinase (ALK) rearrangements are clinically actionable alterations because they guide the use of matched tyrosine kinase inhibitors (TKIs), including agents with central nervous system (CNS) activity [8,9]. Accordingly, accurate identification of targetable EGFR/ALK status is clinically important in NSCLC-BM.

However, molecular profiling remains mainly tissue-based, which is not always feasible for BMs because lesions may be small, multifocal, located in eloquent brain regions, or unsuitable for biopsy [10]. Primary or extracranial tumor results may also be imperfect surrogates because molecular discordance between primary and metastatic sites occurs in 30–53% of cases [11]. Liquid biopsy is useful but may have limited sensitivity in CNS-isolated disease [12,13]. These constraints create a need for noninvasive tools to support molecular diagnosis in BMs when lesion-level testing is unavailable or delayed.

MRI-based artificial intelligence (AI) offers a potential route for noninvasive molecular prediction. Radiomics extracts handcrafted imaging features, whereas deep learning (DL) learns imaging representations directly from MRI data [14]. Although imaging-based mutation prediction has been explored in lung cancer [15,16], studies in NSCLC-BM remain limited and have often focused on radiomics, single-sequence MRI, or EGFR status alone [17,18,19,20,21]. Because EGFR and ALK alteration rates vary across populations, particularly between Western and Asian patients, models developed in one population may not generalize well to another [22,23]. Therefore, a multimodal model integrating complementary MRI sequences and validated across independent cohorts is needed.

Transformer-based models provide a promising alternative [24]. Self-attention captures global context and flexible interactions, and in multimodal imaging it enables adaptive fusion: the model can learn how much each sequence matters and how signals interact across scales. By enabling token-level cross-sequence exchange and dynamic weighting, Transformers can address key weaknesses of naïve fusion and improve robustness when discriminative cues are subtle [25,26]. To the best of our knowledge, Transformer-based cross-scale fusion strategies have not yet been applied to the task of driver gene prediction in NSCLC-BM.

Therefore, we developed and externally validated DriverNet, a clinical and MRI-based framework for noninvasive prediction of targetable driver-positive NSCLC-BM. The primary endpoint was EGFR-mutant and/or ALK-rearranged status (EGFR/ALK+), defined as a clinically oriented molecular triage endpoint. We compared clinical-only, radiomics, DL, and multimodal fusion models using routine pretreatment clinical data, T1CE, and T2-FLAIR MRI and further assessed interpretability and exploratory prognostic stratification.

## 2. Materials and Methods

### 2.1. Ethics Declaration

The study was conducted in accordance with the Declaration of Helsinki. The protocol was approved by the Institutional Review Boards of the Cancer Hospital, Chinese Academy of Medical Sciences (Ethic ID: 26/049-5774; approved 17 January 2026) and the First Affiliated Hospital of Anhui Medical University (Ethic ID: 20230224; approved 26 November 2025), with informed consent waived due to the study’s retrospective nature.

### 2.2. Patients’ Clinical Baseline Information

This multicenter retrospective study analyzed 374 patients with pathologically confirmed NSCLC-BM across three independent cohorts (June 2019–October 2025). Data were pooled from three sources: Center 1 (*n* = 224; Cancer Hospital, CAMS), Center 2 (*n* = 54; First Affiliated Hospital of Anhui Medical University), and Center 3 (*n* = 96; Western cohort from TCIA [27]). Regarding clinical outcomes, complete OS data were available for 161 patients in Center 1 and all 54 patients in Center 2. The list of patients included from TCIA is detailed in the Supplementary Material S2. The Center 1 development cohort was split into training and internal validation subsets strictly (8:2) at the patient level before feature selection, model fitting, or slice extraction. Center 2 and Center 3 were used for external testing.

**Figure 1:**
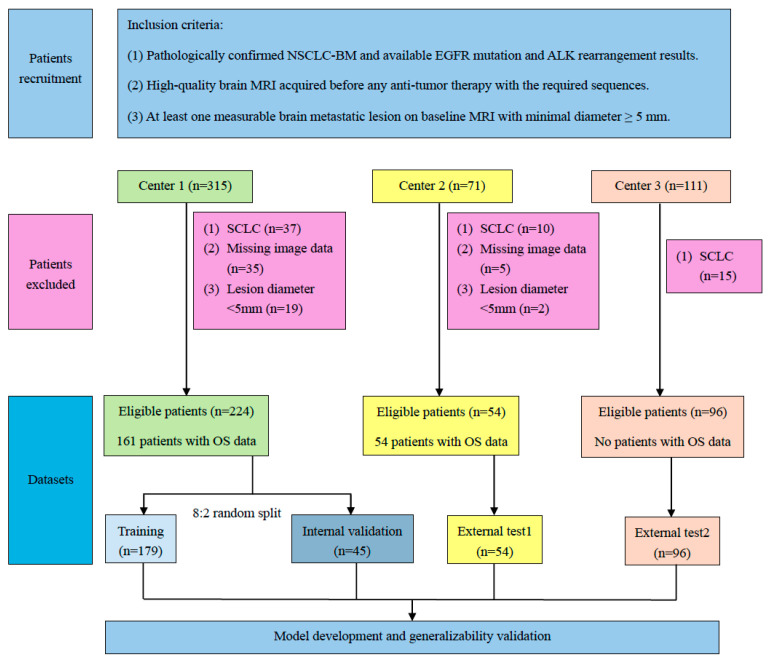
Flowchart of patient selection, cohort allocation, and availability of survival data across the three study centers. SCLC, small-cell lung cancer; OS, overall survival.

Patients were eligible if they met all of the following criteria: [1] pathologically confirmed NSCLC-BM with available EGFR mutation or ALK rearrangement results; [2] pretreatment brain MRI performed before surgery, radiotherapy, chemotherapy, immunotherapy, or targeted therapy, including both T1CE and T2-FLAIR; and [3] at least one measurable brain metastatic lesion on baseline MRI with a minimum diameter of 5 mm. Exclusion criteria were: [1] prior treatment likely to alter lesion appearance before baseline MRI; [2] history of another primary malignancy or major non-neoplastic central nervous system disorder that could confound imaging interpretation; [3] invalid or missing molecular results; and [4] incomplete MRI, severe imaging artifacts, non-measurable lesions, or incomplete essential clinical records. The patient selection workflow is shown in Figure 1.

### 2.3. EGFR and ALK Status Testing

EGFR mutation and ALK rearrangement status were determined in the molecular pathology laboratories of the participating centers using routine clinically validated assays. All molecular analyses were performed on brain metastasis specimens obtained by surgical resection or stereotactic biopsy. EGFR alterations were assessed by targeted next-generation sequencing according to local clinical protocols and reported as EGFR-mutant or EGFR-wild type. ALK status was evaluated by immunohistochemistry and/or fluorescence in situ hybridization, with next-generation sequencing used when applicable, according to center-specific validated criteria, and was classified as ALK-positive or ALK-negative. Molecular results were extracted from pathology reports and electronic medical records; if repeated testing was available, the result closest to the baseline pretreatment MRI was used for labeling. Finally, patients were classified as EGFR/ALK-positive (EGFR/ALK+) or EGFR/ALK-negative (EGFR/ALK−).

### 2.4. MRI Image Preprocessing and Segmentation

All preoperative MRI scans were acquired using 1.5T or 3.0T scanners (MRI data from Center 1 were acquired using scanners manufactured by GE HealthCare, Chicago, IL, USA. MRI data from Center 2 were acquired using scanners manufactured by Philips Healthcare, Best, the Netherlands. MRI data from Center 3 were obtained from The Cancer Imaging Archive (TCIA)), with detailed scanning parameters provided in Supplementary Material S1, Table S7. T1CE images were obtained after intravenous gadolinium-based contrast administration according to each center’s routine clinical protocol; because of the retrospective multicenter design, contrast agent type, dose, and timing were not fully standardized across centers. To decrease the scanner variability, this study used axial T1CE and T2-FLAIR sequences exported from the PACS for model development. Image data were preprocessed using a standardized pipeline that included N4 bias field correction, resampling to an isotropic voxel size of 1 × 1 × 1 mm^3^, and rigid registration of T2-FLAIR images to the T1CE reference space. After that, sequence-wise z-score intensity normalization was applied before radiomics feature extraction and DL input generation.

Tumor segmentation was performed using ITK-SNAP (version 3.8.0, https://www.itksnap.org). The region of interest (ROI) was manually delineated on pretreatment T1CE images for the surgically resected lesion. In patients with multiple brain metastases, the largest surgically resected enhancing lesion was defined as the index lesion for segmentation and model development. Initial segmentation was performed by a neurosurgeon/neuroradiologist with 5 years of experience (Hongliang Mao), and all ROIs were subsequently reviewed and validated by a senior neuroradiologist with more than 20 years of experience (Hongmei Zhang). To evaluate the reliability, a subset of 50 randomly selected cases was independently segmented by both readers. The inter-rater Dice similarity coefficient (DSC) was 0.92 ± 0.14, indicating high consistency in tumor boundary definition.

### 2.5. Radiomics Feature Extraction and Selection

Radiomic feature extraction was performed using the PyRadiomics library (https://pyradiomics.readthedocs.io/) on the 3D segmented ROIs for each MRI sequence. Texture features were calculated from the full 3D ROIs. For each sequence, handcrafted radiomic features were computed and categorized into seven groups: (i) shape, (ii) first-order intensity statistics, (iii) gray-level co-occurrence matrix (GLCM), (iv) gray-level dependence matrix (GLDM), (v) gray-level run-length matrix (GLRLM), (vi) gray-level size zone matrix (GLSZM), and (vii) neighboring gray-tone difference matrix (NGTDM). In total, 1198 radiomic features were extracted per sequence.

Radiomic features underwent a three-step selection process to reduce dimensionality and mitigate overfitting. First, univariate statistical tests identified features significantly associated with EGFR/ALK status. Second, pairwise correlation analysis was performed to eliminate redundancy, retaining only one representative from feature pairs with |r| > 0.9. Finally, the remaining features were input into a sequence-specific LASSO logistic regression, with the optimal penalty parameter determined via cross-validation. This pipeline yielded 11 and 5 non-zero coefficient features for the T1CE and T2-FLAIR models, respectively (Figure S1), which served as inputs for the downstream classification of EGFR and ALK status.

### 2.6. Radiomics Unimodal Model Development

Two single-modality radiomics models were developed independently from T1CE and T2-FLAIR selected features (Rad T1CE and Rad T2-FLAIR). A panel of machine-learning algorithms, including AdaBoost, k-nearest neighbors (KNN), LightGBM, logistic regression (LR), naïve Bayes (NB), and random forest (RF), were evaluated. Model training and hyperparameter optimization were conducted exclusively in the training set using five-fold cross-validation with grid search. Based on cross-validated performance, AdaBoost achieved the best overall results for both Rad T1CE and Rad T2-FLAIR and was therefore selected to build the final unimodal radiomics models (Supplementary Material S1, Tables S1 and S2, Figures S2 and S3).

### 2.7. 2D and Multichannel 2.5D DL Unimodal Model Development

For 2D DL modeling, the axial slice containing the largest tumor ROI was selected from each sequence. For 2.5D modeling, the slice with the largest ROI and adjacent slices within ±3 mm were combined to form a three-channel pseudo-RGB input, preserving limited through-plane contextual information while maintaining computational efficiency. Each input was resized to 224 × 224 × 3.

Five DL architectures were evaluated: ResNet50, ResNet101, DenseNet121, MobileNetV2, and CrossFormer. Training was performed using stochastic gradient descent (SGD) with an initial learning rate of 0.001, momentum of 0.9, and weight decay of 1 × 10^−4^, and all networks were initialized with ImageNet-pretrained weights [28]. A fixed batch size of 32 was used to facilitate balanced batch sampling. Because the training prevalence of EGFR/ALK+ cases was not extremely imbalanced, no additional class weighting was applied in the primary DL experiments. Data augmentation was applied only to the training set and included random horizontal flipping, small-angle rotation, translation, and intensity jittering; no augmentation was applied to validation or external test sets. Early stopping was triggered when the validation AUC did not improve for 20 consecutive epochs, and the model with the best validation AUC was retained. Random seeds were fixed for data splitting and model initialization to improve reproducibility. The models output raw probabilistic prediction scores. DenseNet121 demonstrated the best performance and was therefore adopted as the primary model (Supplementary Material S1, Tables S3–S6, Figures S4–S7). This yielded four unimodal DL models: 2D T1CE, 2D T2-FLAIR, 2.5D T1CE, and 2.5D T2-FLAIR.

### 2.8. Unimodal Deep Feature Extraction

With DenseNet121 as the primary backbone, the pre-trained unimodal networks served as feature extractors. Specifically, the final classification head was removed to extract 1024-dimensional deep embeddings from the global average pooling layer. To facilitate multimodal integration, principal component analysis (PCA) was fitted on the training set and applied to all splits using the same transformation, reducing each modality-specific feature vector from 1024 to 64 dimensions. These reduced embeddings were used for multimodal fusion.

### 2.9. Stacking and Multimodal Fusion Model Development

**Figure 2:**
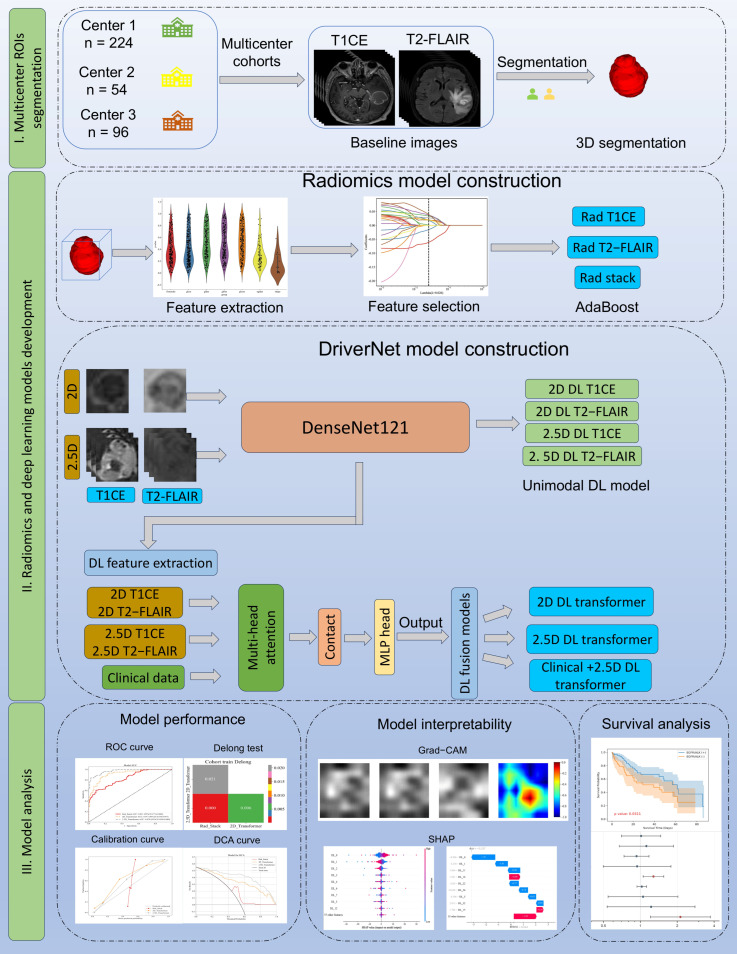
Overview of the DriverNet workflow. Pretreatment T1CE and T2-FLAIR MR images were segmented and used for radiomics and deep-learning model development. DenseNet121-derived 2D/2.5D features were integrated by Transformer-based multimodal fusion, followed by model evaluation, interpretability analyses, and survival assessment.

Two distinct fusion strategies were employed. For radiomics, the output probabilities from sequence-specific AdaBoost models were integrated via a stacking classifier to construct the Rad-stack model. For DL, reduced feature embeddings from T1CE and T2-FLAIR were integrated using a Transformer-based framework with a multilayer perceptron (MLP) head. Each sequence-specific 64-dimensional embedding was first projected into a shared token space. The Transformer fusion module consisted of two encoder layers with multi-head self-attention, four attention heads, feed-forward hidden dimensions of 128, layer normalization, GELU activation, dropout regularization (dropout rate 0.1), and 30 epochs. The fused token representation was then passed to an MLP classifier to generate the final EGFR/ALK probability. This architecture was designed to capture cross-sequence and cross-scale interactions, offering a more robust exploitation of complementary MRI information than conventional concatenation or late averaging. Both 2D and 2.5D Transformer variants were developed. The final model, DriverNet (Figure 2), integrates clinical variables with 2.5D imaging features to provide the comprehensive prediction of EGFR/ALK status in NSCLC-BM.

### 2.10. Model Comparison and Interpretability

To assess model performance, we evaluated accuracy, AUC, sensitivity, specificity, precision, positive predictive value (PPV), negative predictive value (NPV), and Brier score. The predictive capabilities of the models were further assessed using receiver operating characteristic (ROC) curves, DCA, and calibration curves. A clinical-only model was developed using the clinical variables selected from multivariable logistic regression and was compared directly with imaging-only and clinical-imaging models to quantify the incremental contribution of MRI features beyond conventional clinical predictors.

To visualize image regions contributing to model predictions, gradient-weighted class activation mapping (Grad-CAM) was generated from the last convolutional layer of the deep learning models. For the Transformer fusion models, Shapley Additive exPlanations (SHAP) were used to assess both global and individual-level feature contributions.

### 2.11. Prognostic Value Assessment of Transformer Fusion Model

Using the predicted labels from the DriverNet model, patients from Center 1 and Center 2 with complete follow-up were stratified into two groups based on their predicted molecular status (EGFR/ALK+ vs. EGFR/ALK−). To evaluate the prognostic relevance of these model-derived labels, Kaplan–Meier survival analysis and log-rank tests were performed. Exploratory multivariable Cox regression was also performed to adjust for available established prognostic factors.

### 2.12. Statistical Analysis

Statistical analysis was performed using Python (version 3.7) with the scikit-learn package (version 0.18). For comparisons between categorical variables, the chi-square test or Fisher’s exact test was applied, while for continuous variables, the Mann–Whitney U-test or independent t-test was used, depending on data distribution. To statistically compare the models, we performed the DeLong test to assess differences in AUC between the models. All statistical tests were two-sided, and *p* < 0.05 was considered statistically significant.

## 3. Results

### 3.1. Patient Baseline Characteristics Analysis

A total of 374 patients with pathologically confirmed NSCLC-BM were enrolled. The primary cohort from Center 1 (*n* = 224) was randomly partitioned into training (*n* = 179) and internal validation (*n* = 45) sets at an 8:2 ratio. Two independent external cohorts were used for test 1 (*n* = 54) and test 2 (*n* = 96). The prevalence of EGFR/ALK+ was 55.3%, 57.8%, 42.6%, and 12.5% across the training, internal validation, test 1, and test 2 cohorts, respectively. This marked decline in prevalence in test 2 highlights a significant shift in prior probability across diverse populations. Comparative analysis revealed significant baseline differences between the training and testing cohorts regarding age, gender, Graded Prognostic Assessment at brain metastasis diagnosis (GPA at BM Dx), and EGFR status (*p* < 0.05; Table 1).

**Figure 3:**
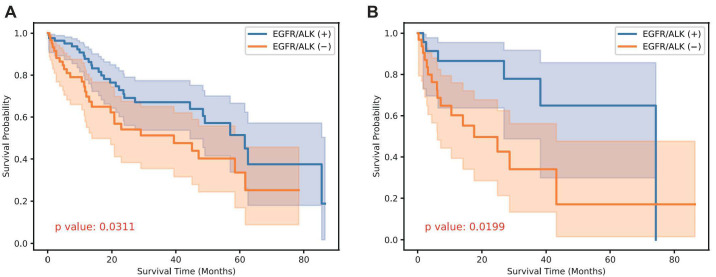
Kaplan–Meier survival curves comparing OS between EGFR/ALK+ and EGFR/ALK− patients in Center 1 (**A**) and Center 2 (**B**). EGFR/ALK+ status was associated with significantly better OS in both centers.

Survival analysis of matched data (Center 1: *n* = 161; Center 2: *n* = 54) confirmed that EGFR/ALK+ status was significantly associated with superior OS in both centers (Figure 3), underscoring its prognostic significance. Furthermore, univariate and multivariate logistic regression identified four clinical features as independent predictors of EGFR/ALK status: gender (*p* < 0.001), Karnofsky Performance Status at brain metastasis diagnosis (KPS at BM Dx) (*p* = 0.027), size of the dominant lesion (*p* = 0.002), and adenocarcinoma histology (*p* < 0.001) (Table 2). These variables were subsequently incorporated into the development of the integrated clinical-imaging models.

### 3.2. Performance of Unimodal Radiomics and DL Models

Unimodal radiomics models demonstrated moderate performance, with Rad T1CE and Rad T2-FLAIR achieving internal validation AUCs of 0.868 (95% CI: 0.768–0.969) and 0.888 (0.791–0.984), respectively. However, both models showed limited generalizability in external cohorts, with AUCs ranging from 0.572 to 0.709. Detailed ROC analyses, calibration results, DCA, and classifier comparisons are shown in Supplementary Material S1, Figures S2 and S3, Tables S1 and S2.

In contrast, DL models, particularly those utilizing DenseNet121, consistently outperformed radiomics. The 2.5D configuration proved superior to 2D, providing enhanced stability across diverse datasets. Specifically, 2.5D T1CE achieved robust AUCs of 0.921 (0.884–0.958) in training and maintained high performance in external test 2 (AUC: 0.867, 0.766–0.968). Similarly, 2.5D T2-FLAIR exhibited excellent discriminative power in external testing, reaching AUCs of 0.938 (0.875–1.000) and 0.874 (0.784–0.964) in test 1 and test 2, respectively. These results indicate that DL-based 2.5D features are more resilient to the prior probability shifts encountered in different ethnic populations. Full performance results for the candidate DL models are shown in Supplementary Material S1, Figures S4–S7, Tables S3–S6.

### 3.3. Multimodal Fusion and Model Integration

We next constructed and compared a clinical-only model, a radiomics stacking model (Rad-stack), a 2D Transformer fusion model, and a 2.5D Transformer fusion model. The clinical-only model was developed from the selected clinical predictors, the Rad-stack model combined output probabilities from unimodal radiomics models into AdaBoost classifiers, and the Transformer fusion models integrated modality-specific deep features from T1CE and T2-FLAIR using a multi-head attention framework.

**Figure 4:**
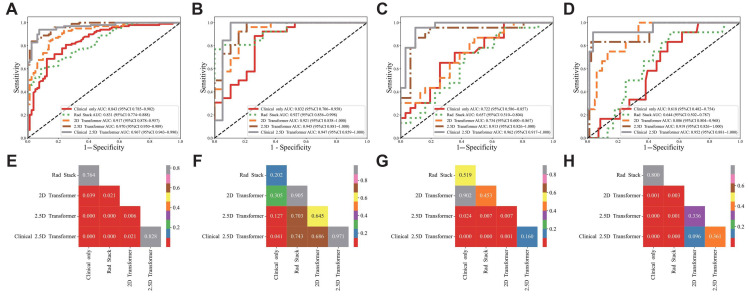
Performance comparison of multimodal fusion models. (**A**–**D**) ROC curves of the Clinical-only, Rad-stack, 2D Transformer, 2.5D Transformer, and Clinical + 2.5D Transformer models in the training cohort (**A**), internal validation cohort (**B**), external test 1 (**C**), and external test 2 (**D**). (**E**–**H**) DeLong tests comparing AUCs among models in the corresponding cohorts. ROC, receiver operating characteristic; AUC, area under the curve.

Among the imaging-based models, the 2.5D Transformer architecture emerged as the superior imaging-only framework, achieving robust and consistent performance across all cohorts, with AUCs of 0.970 (95% CI: 0.950–0.989) in training, 0.945 (0.881–1.000) in internal validation, and 0.913 (0.826–1.000) and 0.919 (0.826–1.000) in the external test sets. The clinical-only model achieved AUCs of 0.843, 0.832, 0.722, and 0.618 in the training, internal validation, test 1, and test 2 cohorts, respectively, indicating that clinical predictors alone carried useful information but generalized less well than the 2.5D imaging fusion model in the external cohorts. In contrast, the Rad-stack and 2D Transformer models exhibited lower discriminative power, particularly in external validation (Figure 4A–D).

**Figure 5:**
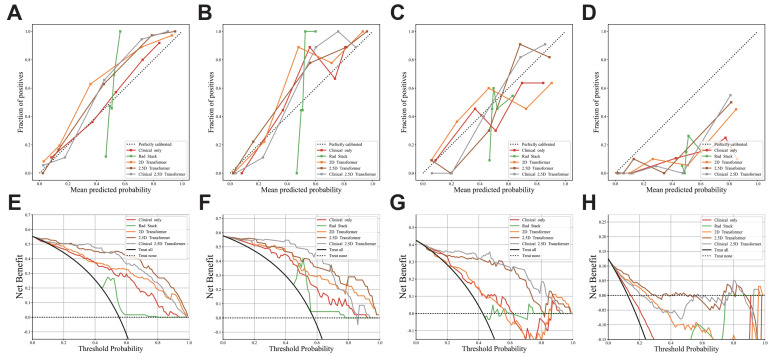
Calibration and decision curve analyses of multimodal fusion models. Calibration curves (**A**–**D**) and DCA curves (**E**–**H**) are shown for the training cohort (**A**,**E**), internal validation cohort (**B**,**F**), external test 1 (**C**,**G**), and external test 2 (**D**,**H**), demonstrating calibration and comparative clinical net benefit among the Clinical-only, Rad-stack, 2D Transformer, 2.5D Transformer, and Clinical + 2.5D Transformer models. DCA, decision curve analysis.

To further optimize predictive accuracy, clinical variables were integrated into the 2.5D Transformer framework, yielding the final clinical + 2.5D Transformer fusion model (DriverNet). This integrated approach achieved the highest overall performance, with AUCs of 0.967 (0.943–0.990) in training, 0.947 (0.859–1.000) in validation, and 0.962 (0.917–1.000) and 0.952 (0.881–1.000) in test 1 and test 2, respectively. Compared with the clinical-only model, DriverNet showed improved discrimination in both external cohorts and lower Brier scores in test 1, supporting incremental predictive value from MRI-derived features. Pairwise DeLong tests confirmed that this integrated model significantly outperformed both the unimodal and imaging-only fusion models (*p* < 0.05; Figure 4E–H). Furthermore, calibration curves showed good agreement between predicted and observed probabilities, and DCA underscored its superior clinical net benefit across a broad range of threshold probabilities (Figure 5). Detailed metrics of models are summarized in Table 3.

### 3.4. Interpretability Analyses

**Figure 6:**
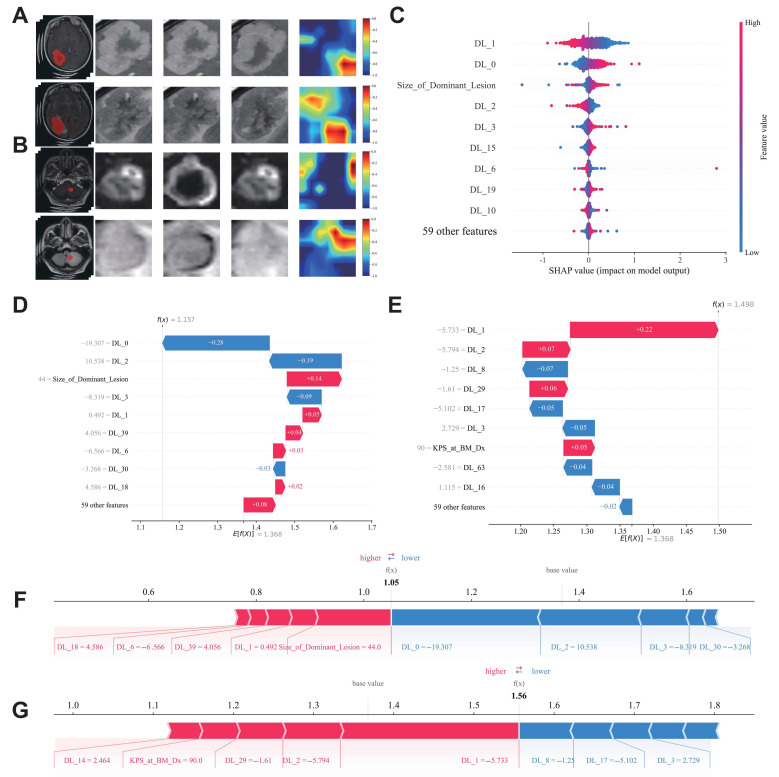
Interpretability analysis of the Clinical + 2.5D CNN–Transformer model. (**A**,**B**) Representative T1CE and T2-FLAIR images with Grad-CAM heatmaps for correctly predicted EGFR/ALK− (**A**) and EGFR/ALK+ (**B**) cases. (**C**) Global SHAP summary plot. (**D**,**E**) SHAP waterfall plots for representative EGFR/ALK− (**D**) and EGFR/ALK+ (**E**) predictions. (**F**,**G**) SHAP force plots visualizing feature-level contributions in the corresponding negative and positive cases. Grad-CAM, gradient-weighted class activation mapping; SHAP, SHapley Additive exPlanations.

To examine the plausibility of model predictions, we performed Grad-CAM and SHAP analyses for the DriverNet model. Grad-CAM heatmaps revealed that the DenseNet121 backbone focused not only on the enhancing lesion but also on sequence-specific regions. On T1CE, the model primarily highlighted the tumor boundary and adjacent peritumoral areas, while on T2-FLAIR, it emphasized the tumor core (Figure 6A,B). This sequence-specific attention pattern is compatible with clinical observation, as the enhancing rim on T1CE often corresponds to regions of active neovascularization and infiltrative growth, while T2-FLAIR more prominently depicts the tumor core and its internal necrotic or solid components.

Additionally, SHAP analysis quantified the contribution of individual features to model predictions. Notably, the size of the dominant lesion had a significant impact on model performance, alongside deep features from the MRI sequences (Figure 6C). SHAP also showed how specific features such as lesion size influenced predictions for both EGFR/ALK− (Figure 6D,F) and EGFR/ALK+ (Figure 6E,G) cases.

### 3.5. Model-Derived Prognostic Stratification

**Figure 7:**
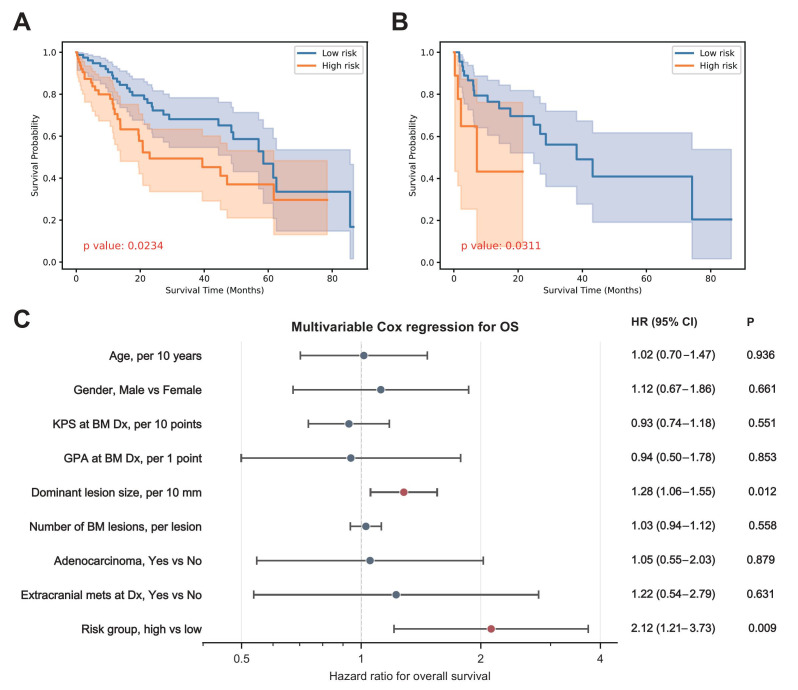
Exploratory prognostic stratification using DriverNet model-derived labels. Kaplan–Meier survival curves stratified by model-derived risk groups in Center 1 (**A**) and Center 2 (**B**), and multivariable Cox regression analysis adjusting for available conventional prognostic variables in the survival cohort (**C**). OS, overall survival.

Based on the predicted labels from the DriverNet model, the study cohorts were categorized into model-derived risk groups for survival analysis. Log-rank tests demonstrated that OS could be significantly stratified in both Center 1 and Center 2 based on these model-derived labels (*p* = 0.023 and *p* = 0.031, respectively; Figure 7A,B). Exploratory multivariable Cox regression further evaluated the association between model-derived labels and OS after adjustment for available conventional prognostic variables (Figure 7C). These findings suggest that the model-derived classification captures clinically relevant information associated with patient outcome; however, the prognostic role of DriverNet should be considered exploratory and requires prospective validation.

## 4. Discussion

In this multicenter study, we developed and validated DriverNet, a multimodal framework for noninvasive prediction of EGFR/ALK status in patients with NSCLC-BM. Among the evaluated strategies, the clinical + 2.5D CNN–Transformer fusion model showed encouraging performance across the internal and external cohorts. The maintained performance in the Western cohort suggests potential imaging associations with targetable driver status; however, this finding should be interpreted cautiously given the small number of EGFR/ALK-positive cases in that cohort. In addition, model-derived labels were associated with OS in exploratory analyses, providing preliminary evidence for further evaluation of this framework.

From a clinical perspective, the potential value of DriverNet is to provide a research-stage noninvasive complement to lesion-level molecular testing when intracranial genotyping is unavailable, delayed, or potentially unrepresentative of metastatic disease. At this stage, DriverNet should not be interpreted as a tool for directing clinical management or replacing tissue-based genotyping or validated liquid biopsy. Rather, its outputs may be explored in future prospective studies as a probabilistic signal to support molecular triage research. This design allowed us to evaluate whether DriverNet performance remained relatively stable across heterogeneous cohorts. However, because the external cohorts were relatively small and test 2 included only 12 EGFR/ALK-positive cases, these findings should be interpreted with appropriate caution. In addition, the composite EGFR/ALK endpoint also warrants careful interpretation. EGFR-mutant and ALK-rearranged NSCLC are biologically distinct entities and may have different radiographic and microenvironmental phenotypes. We therefore used the EGFR/ALK+ label as a pragmatic targetable-driver triage endpoint rather than as evidence of biological equivalence. Separate ALK-specific modeling was not feasible because ALK-positive cases were few across cohorts, creating substantial class imbalance and unstable estimates. This limitation is clinically important and should be addressed in larger datasets enriched for ALK-rearranged NSCLC-BM.

Compared with previous MRI-based molecular prediction studies in NSCLC-BM, an important difference of DriverNet is that it extends radiomics prediction from a single-marker task to a clinically oriented targetable-driver triage framework. Earlier radiomics studies demonstrated promising performance for EGFR, ALK, or KRAS prediction, such as the cross-validated AUCs of 0.912, 0.915, and 0.985 reported by Chen et al. [17] and the multicenter habitat-based EGFR radiomics model developed by Cao et al. [18]. However, these approaches mainly relied on handcrafted radiomics features and did not evaluate DL-based multisequence fusion. Subsequent DL studies further supported the feasibility of MRI-based EGFR prediction, including the AUC of 0.91 reported by Haim et al. in 59 patients [20], but they remained largely EGFR-centered and did not directly compare radiomics, 2D DL, 2.5D DL, and multimodal fusion strategies. In contrast, DriverNet achieved AUCs of 0.947, 0.962, and 0.952 in the internal validation and two external cohorts, respectively, while integrating clinical variables with T1CE and T2-FLAIR features through a 2.5D Transformer fusion framework. Therefore, our study provides additional evidence by broadening the endpoint to EGFR/ALK targetable-driver triage, systematically benchmarking multiple model families, incorporating calibration assessment with the Brier score, and validating the model across independent cohorts with markedly different driver-positive prevalence. A structured comparison of DriverNet and previously published MRI-based prediction studies is provided in Supplementary Material S1, Table S8.

A central finding of this study is the superiority of the 2.5D CNN–Transformer fusion strategy and the incremental value of MRI-derived information beyond clinical predictors alone. DriverNet incorporated four clinically accessible variables: gender, KPS at BM Dx, size of the dominant lesion, and adenocarcinoma histology, which were significantly associated with EGFR/ALK status in our cohort. This is broadly consistent with previous literature showing that EGFR/ALK+ NSCLC-BM is more frequently associated with adenocarcinoma histology, female sex, and distinct clinicopathologic patterns [29,30,31], supporting the biological and clinical rationale for integrating these variables with imaging features. However, the clinical-only model showed weaker external performance than DriverNet, particularly in the Western cohort, indicating that the integrated model was not driven solely by established clinical predictors. The improved performance of DriverNet likely reflects the combination of richer spatial context and more effective cross-sequence integration. Compared with conventional 2D models, the 2.5D representation preserves limited through-plane continuity by incorporating adjacent slices around the target lesion, thereby capturing spatial heterogeneity while avoiding the computational burden and sample-size demands of full 3D modeling [25,32]. In our framework, each sequence-specific 2.5D input was constructed as a three-channel pseudo-RGB image, enabling direct initialization from ImageNet-pretrained CNN backbones while retaining inter-slice contextual information [28,33,34]. After sequence-specific CNN feature extraction, the Transformer module further enabled adaptive fusion by modeling cross-sequence interactions and long-range dependencies [24,25,26]. This is particularly relevant for T1CE and T2-FLAIR, which reflect distinct yet complementary aspects of tumor biology, including blood–brain barrier disruption, enhancement behavior, edema, and the peritumoral microenvironment [35]. Moreover, despite incorporating multimodal fusion, DriverNet maintained a moderate computational burden relative to several commonly used deep-learning architectures (Supplementary Material S1, Table S9).

An additional observation from our experiments is that CrossFormer did not outperform DenseNet121 as the unimodal backbone. A plausible explanation is that transformer-style vision backbones generally require larger-scale datasets to fully exploit their cross-scale attention advantages [36], whereas our study, although multicenter, still represents a moderate-sized cohort with substantial imaging heterogeneity. Under these conditions, convolutional architectures may provide more stable and sample-efficient feature extraction. In addition, the imaging cues associated with targetable driver-positive status in NSCLC-BM may depend more strongly on localized texture, enhancement boundary, and lesion–parenchyma interface characteristics [19]. Therefore, in this application, the major performance gain appears to derive more from robust multimodal integration than from increasing the complexity of the unimodal feature extractor.

Our results also highlight differences in the external robustness of radiomics and DL approaches. While the radiomics models showed acceptable performance in the development cohort, their performance declined more noticeably in external testing, especially in the Western cohort, consistent with the known sensitivity of handcrafted features to scanner platform, acquisition protocol, preprocessing, and segmentation variability [14,37]. No explicit scanner-level harmonization methods such as ComBat were applied in this study, and contrast-enhanced MRI protocols were not completely standardized across centers. Therefore, the observed external performance should be interpreted in the context of residual acquisition heterogeneity. By contrast, the DL models, particularly the 2.5D CNN–Transformer, were more resilient to such heterogeneity, suggesting that learned hierarchical representations may offer better cross-center generalizability. This is clinically important because the Western external cohort had a substantially lower prevalence of EGFR/ALK+ [22,23], a prior-probability shift that can affect calibration, predictive values, and transportability. The maintained performance of DriverNet under this setting suggests possible phenotype–genotype associations beyond a single development population, but broader prospective validation remains necessary before clinical use can be considered. Such validation is essential for any imaging-based model intended for future clinical translation and is also aligned with current expectations for external validation, transparency, and reproducibility in medical AI research [38,39].

Interpretability analyses further support the face validity of the model. Grad-CAM showed that salient regions were not uniformly distributed across the lesion but instead demonstrated a distinct sequence-specific attention pattern. On T1CE, the model primarily focused on the tumor boundary and adjacent peritumoral regions, whereas on T2-FLAIR it placed greater attention on the tumor core. This pattern is broadly compatible with clinical observation. The enhancing rim on T1CE often corresponds to regions of active neovascularization, blood–brain barrier disruption, and infiltrative tumor growth, while T2-FLAIR more prominently reflects the tumor core together with its internal necrotic or solid components and surrounding tissue response [40]. SHAP complemented these findings by providing both global and case-level explanations of how fused deep features and clinical variables contributed to the final prediction. Notably, beyond the imaging biomarkers, size of the dominant lesion emerged as one of the most influential clinical features, underscoring that lesion burden made a substantial contribution to EGFR/ALK prediction. Nevertheless, Grad-CAM and SHAP do not prove histopathologic causality, and we did not quantitatively evaluate feature-importance stability across cohorts. More broadly, explainable AI approaches are increasingly important in determining whether a biomarker model relies on clinically meaningful signals rather than spurious associations [41].

The prognostic analyses add further translational context to this framework but should not be overinterpreted. In our survival cohorts, EGFR/ALK status was associated with outcome, and model-derived labels stratified OS in both available centers. Exploratory multivariable Cox regression was added to assess whether this association persisted after adjustment for available conventional prognostic factors. These analyses suggest that DriverNet-derived classifications may be associated with prognosis in the available cohorts, but they cannot establish independent prognostic utility in the absence of prospective follow-up, uniform treatment data, and survival data from the TCIA cohort. Future studies may evaluate whether such a model could provide complementary information for pre-treatment molecular triage when tissue-based profiling is incomplete or delayed. MRI-based prediction should not be considered a substitute for molecular diagnostics performed on tissue or validated liquid biopsy specimens.

Several limitations should be acknowledged. First, this was a retrospective multicenter study and was therefore subject to selection bias, residual confounding, center-specific effects, and incomplete treatment information for survival analyses. Second, although the cohort was relatively substantial for an imaging study of NSCLC-BM, the overall sample size remained moderate for DL, particularly after stratification by center and molecular subtype; external test 2 included only 12 EGFR/ALK-positive cases, making confidence intervals and threshold-dependent metrics potentially unstable. Third, the model analyzed a single dominant/index lesion and used only routine T1CE and T2-FLAIR sequences; therefore, it may not fully capture inter-lesional heterogeneity in multifocal BM or potentially informative signals from diffusion, perfusion, or other advanced MRI sequences. Fourth, MRI acquisition protocols and scanner platforms were heterogeneous, no explicit scanner-level harmonization method was applied, and Grad-CAM/SHAP analyses remained descriptive. Broader prospective validation with standardized imaging protocols, balanced molecular subgroups, and lesion-level radiogenomic correlation is required before clinical implementation.

## 5. Conclusions

In conclusion, we developed and externally validated DriverNet as a clinical and MRI-based framework for noninvasive identification of EGFR/ALK status in NSCLC-BM. By integrating routine clinical variables with T1CE and T2-FLAIR features, DriverNet showed encouraging performance across independent cohorts with different mutation prevalence and an exploratory association with prognosis. At this stage, DriverNet should be regarded as a complementary imaging tool rather than a clinically applicable biomarker or a replacement for tissue-based molecular testing. Prospective validation in larger and more molecularly balanced cohorts is required before any clinical implementation can be considered.

## Figures and Tables

**Table 1:** Comparison of baseline characteristics of patients in different cohorts.

	Train	Internal Validation	External Test 1	External Test 2	*p* Value
Age, year (mean ± SD)	60.4 ± 9.2	64.6 ± 8.4	64.3 ± 9.8	65.1 ± 9.1	<0.001
KPS at BM Dx, median [IQR]	80.0 [70.0, 80.0]	80.0 [70.0, 90.0]	75.0 [62.5, 80.0]	80.0 [70.0, 82.5]	0.553
GPA at BM Dx, median [IQR]	2.5 [1.5, 3.0]	2.5 [2.0, 3.0]	2.0 [1.5, 2.5]	1.5 [1.0, 2.5]	<0.001
Size of dominant lesion, cm (mean ± SD)	26.9 ± 13.7	23.2 ± 13.1	27.5 ± 12.9	27.0 ± 11.0	0.317
Number of BM lesions, median [IQR]	1 [1, 3]	1 [1, 2]	1 [1, 2]	2 [1, 4]	0.023
Gender, *n* (%)					0.003
Female	72 (40.2%)	18 (40.0%)	22 (40.7%)	60 (62.5%)	
Male	107 (59.8%)	27 (60.0%)	32 (59.3%)	36 (37.5%)	
Adenocarcinoma, *n* (%)					0.899
No	33 (18.4%)	7 (15.6%)	10 (18.5%)	20 (20.8%)	
Yes	146 (81.6%)	38 (84.4%)	44 (81.5%)	76 (79.2%)	
Extracranial Mets at Dx, *n* (%)					0.381
No	65 (36.3%)	17 (37.8%)	13 (24.1%)	33 (34.4%)	
Yes	114 (63.7%)	28 (62.2%)	41 (75.9%)	63 (65.6%)	
EGFR mutation, *n* (%)					<0.001
No	89 (49.7%)	22 (48.9%)	36 (66.7%)	85 (88.5%)	
Yes	90 (50.3%)	23 (51.1%)	18 (33.3%)	11 (11.5%)	
ALK rearrangement, *n* (%)					0.129
No	170 (95.0%)	42 (93.3%)	49 (90.7%)	95 (99.0%)	
Yes	9 (5.0%)	3 (6.7%)	5 (9.3%)	1 (1.0%)	
EGFR/ALK (+), *n* (%)					<0.001
No	80 (44.7%)	19 (42.2%)	31 (57.4%)	84 (87.5%)	
Yes	99 (55.3%)	26 (57.8%)	23 (42.6%)	12 (12.5%)	

SD, standard deviation; KPS at BM Dx, Karnofsky performance status at brain metastasis diagnosis; GPA at BM Dx, Graded Prognostic Assessment at brain metastasis diagnosis; Extracranial Mets at Dx, extracranial metastases at diagnosis.

**Table 2:** Univariate and multivariate logistic regression analysis for the cohort from Center 1.

		Univariate		Multivariate	
	*n*	OR (95% CI)	*p* Value	OR (95% CI)	*p* Value
Age	224	0.953 (0.924–0.983)	0.002	0.965 (0.917–1.015)	0.163
Gender					
Male	134	0.119 (0.062–0.229)	<0.001	0.148 (0.071–0.307)	<0.001
Female (Reference)	90				
KPS at BM Dx	224	1.025 (1.003–1.047)	0.024	1.036 (1.004–1.068)	0.027
GPA at BM Dx	224	1.103 (0.832–1.461)	0.495	0.863 (0.385–1.935)	0.720
Size of dominant lesion	224	1.010 (0.991–1.030)	0.312	1.046 (1.017–1.076)	0.002
Number of BM lesions	224	1.118 (1.010–1.238)	0.032	1.084 (0.947–1.242)	0.241
Adenocarcinoma					
Yes	184	17.286 (5.888–50.745)	<0.001	13.752 (4.099–46.135)	<0.001
No (Reference)	40				
Extracranial Mets at Dx					
Yes	142	1.448 (0.838–2.503)	0.185	1.057 (0.337–3.320)	0.924
No (Reference)	82				

OR, odds ratio; CI, confidence interval; LR, likelihood ratio; KPS at BM Dx, Karnofsky performance status at brain metastasis diagnosis; GPA at BM Dx, Graded Prognostic Assessment at brain metastasis diagnosis; Extracranial Mets at Dx, extracranial metastases at diagnosis.

**Table 3:** Performance of multimodal fusion models.

Model	Accuracy	AUC	95% CI	Sensitivity	Specificity	PPV	NPV	Brier Score	Cohort
Clinical only	0.771	0.843	0.785–0.902	0.687	0.875	0.872	0.693	0.162	Train
Rad-stack	0.743	0.831	0.774–0.888	0.606	0.912	0.896	0.652	0.231	
2D Transformer	0.855	0.917	0.878–0.957	0.869	0.837	0.869	0.837	0.139	
2.5D Transformer	0.899	0.970	0.950–0.989	0.838	0.975	0.976	0.830	0.085	
Clinical + 2.5D Transformer	0.933	0.967	0.943–0.990	0.939	0.925	0.939	0.925	0.094	
Clinical only	0.822	0.832	0.706–0.958	0.885	0.737	0.821	0.824	0.164	Internal validation
Rad-stack	0.867	0.927	0.856–0.998	0.769	1.000	1.000	0.760	0.222	
2D Transformer	0.889	0.921	0.838–1.000	0.923	0.842	0.889	0.889	0.127	
2.5D Transformer	0.911	0.945	0.881–1.000	1.000	0.789	0.867	1.000	0.100	
Clinical + 2.5D Transformer	0.956	0.947	0.859–1.000	1.000	0.895	0.929	1.000	0.085	
Clinical only	0.704	0.722	0.586–0.857	0.652	0.742	0.652	0.652	0.220	Test 1
Rad-stack	0.648	0.657	0.510–0.804	0.739	0.581	0.567	0.750	0.245	
2D Transformer	0.667	0.734	0.600–0.867	0.870	0.516	0.571	0.842	0.227	
2.5D Transformer	0.889	0.913	0.826–1.000	0.870	0.903	0.870	0.903	0.118	
Clinical + 2.5D Transformer	0.926	0.962	0.917–1.000	0.957	0.903	0.880	0.966	0.096	
Clinical only	0.521	0.618	0.482–0.754	0.833	0.476	0.185	0.952	0.349	Test 2
Rad-stack	0.510	0.644	0.502–0.787	0.917	0.452	0.193	0.974	0.268	
2D Transformer	0.708	0.886	0.804–0.968	1.000	0.667	0.300	1.000	0.154	
2.5D Transformer	0.958	0.919	0.826–1.000	0.833	0.976	0.833	0.976	0.098	
Clinical + 2.5D Transformer	0.958	0.952	0.881–1.000	0.917	0.964	0.786	0.988	0.120	

AUC, area under the curve; CI, confidence interval; PPV, positive predictive value; NPV, negative predictive value.

## Data Availability

The data used in this study were collected from three participating centers and contain identifiable clinical and imaging information from patients with non-small-cell lung cancer brain metastases. Owing to patient privacy concerns and institutional ethical restrictions, these data are not publicly available. De-identified data may be made available from the corresponding author upon reasonable request and subject to approval by the participating institutions and relevant ethics committees. The analyses were implemented using the Onekey AI platform (https://github.com/OnekeyAI-Platform, accessed on 1 May 2026).

## Supplementary Materials

The following supporting information can be downloaded at: https://www.mdpi.com/article/10.3390/diagnostics16131988/s1, Supplementary Material S1 includes additional model-performance tables, supplementary figures, and MRI acquisition parameters; Supplementary Material S2 includes the list of TCIA patients used in the external cohort.

## Abbreviations

The following abbreviations are used in this manuscript:

AI	artificial intelligence
ALK	anaplastic lymphoma kinase
AUC	area under the curve
BM	brain metastasis
CI	confidence interval
CNN	convolutional neural network
DCA	decision curve analysis
DL	deep learning
DSC	Dice similarity coefficient
EGFR	epidermal growth factor receptor
GPA	Graded Prognostic Assessment
KPS	Karnofsky Performance Status
MRI	magnetic resonance imaging
NPV	negative predictive value
NSCLC	non-small-cell lung cancer
OR	odds ratio
OS	overall survival
PACS	picture archiving and communication system
PCA	principal component analysis
PPV	positive predictive value
ROI	region of interest
ROC	receiver operating characteristic
SHAP	SHapley Additive exPlanations
TCIA	The Cancer Imaging Archive
TKI	tyrosine kinase inhibitor
