# Supplementary material for: DriverNet: A Clinical and MRI-Based Framework for Noninvasive Pre-Treatment Molecular Triage in NSCLC Brain Metastases
Source: Diagnostics (Basel). 2026 Jun 26;16(13):1988. doi: 10.3390/diagnostics16131988 (PMC13359782; doi:10.3390/diagnostics16131988)
Supplement: Supplementary file 1 [file diagnostics-16-01988-s001.zip › Supplementary materials S1.pdf]

Supplementary material S1

Table S1: Performances of six ML methods in the Rad T1CE unimodal model.

Table S2: Performances of six ML methods in the Rad FLAIR unimodal model.

Table S3: Performances of five DL methods in the 2D T1CE unimodal model.

Table S4: Performances of five DL methods in the 2D T2-FLAIR unimodal model.

Table S5: Performances of five DL methods in the 2.5D T1CE unimodal model.

Table S6: Performances of five DL methods in the 2.5D T2-FLAIR unimodal model.

Table S7: MRI acquisition parameters for T1CE and T2-FLAIR across three centers.

Table S8: Comparison of DriverNet with previously published MRI-based molecular prediction studies in NSCLC brain metastases.

Table S9: Computational complexity of the evaluated deep-learning architectures and the final DriverNet framework.

Figure S1. Radiomics feature distribution and LASSO-based feature selection.

Figure S2. Performance comparison of six ML classifiers for the Rad T1CE model.

Figure S3. Performance comparison of six ML classifiers for the Rad T2-FLAIR model.

Figure S4. Performance comparison of five DL classifiers for the 2D T1CE model.

Figure S5. Performance comparison of five DL classifiers for the 2D T2-FLAIR model.

Figure S6. Performance comparison of five DL classifiers for the 2.5D T1CE model.

Figure S7. Performance comparison of five DL classifiers for the 2.5D T2-FLAIR model.

Table S1: Performances of six ML methods in the Rad T1CE unimodal model.

Model	Accuracy	AUC	95% CI	Sensitivity	Specificity	PPV	NPV	Cohort
AdaBoost	0.732	0.815	0.755 - 0.875	0.586	0.912	0.892	0.640	
KNN	0.698	0.800	0.739 - 0.861	0.556	0.875	0.846	0.614	
LightGBM	0.615	0.628	0.559 - 0.696	0.505	0.750	0.714	0.550	Train
LR	0.637	0.706	0.631 - 0.782	0.485	0.825	0.774	0.564	
NB	0.637	0.669	0.589 - 0.748	0.596	0.687	0.702	0.579	
RF	0.743	0.811	0.750 - 0.873	0.717	0.775	0.798	0.689	
AdaBoost	0.733	0.868	0.768 - 0.969	0.538	1.000	1.000	0.613	
KNN	0.778	0.838	0.726 - 0.951	0.846	0.684	0.786	0.765	
LightGBM	0.689	0.731	0.633 - 0.829	0.462	1.000	1.000	0.576	Internal validation
LR	0.800	0.812	0.680 - 0.944	0.808	0.789	0.840	0.750	
NB	0.733	0.791	0.653 - 0.930	0.654	0.842	0.850	0.640	
RF	0.911	0.970	0.930 - 1.000	0.923	0.895	0.923	0.895	
AdaBoost	0.611	0.648	0.501 - 0.795	0.957	0.355	0.524	0.917	
KNN	0.574	0.543	0.390 - 0.696	0.870	0.355	0.500	0.786	
LightGBM	0.593	0.561	0.437 - 0.686	0.348	0.774	0.533	0.615	Test 1
LR	0.574	0.523	0.367 - 0.680	1.000	0.258	0.500	1.000	
NB	0.611	0.588	0.432 - 0.743	0.522	0.677	0.545	0.656	
RF	0.500	0.517	0.359 - 0.675	0.957	0.161	0.458	0.833	
AdaBoost	0.500	0.709	0.568 - 0.850	0.917	0.440	0.190	0.974	
KNN	0.698	0.712	0.557 - 0.866	0.750	0.690	0.257	0.951	
LightGBM	0.125	0.488	0.400 - 0.576	1.000	0.000	0.125	0.000	Test 2
LR	0.521	0.621	0.471 - 0.771	0.917	0.464	0.196	0.975	
NB	0.708	0.605	0.413 - 0.797	0.667	0.714	0.250	0.937	
RF	0.771	0.556	0.374 - 0.737	0.417	0.821	0.250	0.908	

AUC, Area under curve; CI, Confidence interval; PPV, Positive predictive value; NPV, Negative predictive value; KNN, K-nearest neighbors; LR, Logistic regression; NB, Naïve Bayes; RF, Random forest.

Table S2: Performances of six ML methods in the Rad T2-FLAIR unimodal model.

Model	Accuracy	AUC	95% CI	Sensitivity	Specificity	PPV	NPV	Cohort
AdaBoost	0.709	0.750	0.680 - 0.821	0.737	0.675	0.737	0.675	Train
KNN	0.709	0.752	0.685 - 0.820	0.768	0.637	0.724	0.689	
LightGBM	0.615	0.628	0.559 - 0.696	0.505	0.750	0.714	0.550	
LR	0.631	0.654	0.573 - 0.735	0.515	0.775	0.739	0.564	
NB	0.637	0.632	0.550 - 0.714	0.667	0.600	0.673	0.593	
RF	0.715	0.778	0.710 - 0.847	0.667	0.775	0.786	0.653	
AdaBoost	0.844	0.888	0.791 - 0.984	0.962	0.684	0.806	0.929	Internal validation
KNN	0.756	0.825	0.709 - 0.941	0.808	0.684	0.778	0.722	
LightGBM	0.689	0.731	0.633 - 0.829	0.462	1.000	1.000	0.576	
LR	0.800	0.856	0.737 - 0.976	0.808	0.789	0.840	0.750	
NB	0.822	0.822	0.689 - 0.954	0.846	0.789	0.846	0.789	
RF	0.733	0.849	0.742 - 0.957	0.538	1.000	1.000	0.613	
AdaBoost	0.611	0.645	0.497 - 0.793	0.957	0.355	0.524	0.917	Test 1
KNN	0.574	0.588	0.440 - 0.737	0.957	0.290	0.500	0.900	
LightGBM	0.593	0.561	0.437 - 0.686	0.348	0.774	0.533	0.615	
LR	0.556	0.518	0.360 - 0.675	0.522	0.581	0.480	0.621	
NB	0.630	0.570	0.408 - 0.732	0.522	0.710	0.571	0.667	
RF	0.519	0.470	0.313 - 0.626	0.826	0.290	0.463	0.692	
AdaBoost	0.448	0.572	0.432 - 0.713	0.917	0.381	0.175	0.970	Test 2
KNN	0.781	0.589	0.398 - 0.780	0.417	0.833	0.263	0.909	
LightGBM	0.125	0.488	0.400 - 0.576	1.000	0.000	0.125	0.000	
LR	0.740	0.557	0.366 - 0.747	0.500	0.774	0.240	0.915	
NB	0.542	0.619	0.476 - 0.762	0.917	0.488	0.204	0.976	
RF	0.760	0.585	0.426 - 0.744	0.500	0.798	0.261	0.918	

AUC, Area under curve; CI, Confidence interval; PPV, Positive predictive value; NPV, Negative predictive value; KNN, K-nearest neighbors; LR, Logistic regression; NB, Naïve Bayes; RF, Random forest.

Table S3: Performances of five DL methods in the 2D T1CE unimodal model.

Model	Accuracy	AUC	95% CI	Sensitivity	Specificity	PPV	NPV	Cohort
ResNet50	0.665	0.700	0.624 - 0.777	0.657	0.675	0.714	0.614	
ResNet101	0.642	0.623	0.541 - 0.705	0.646	0.637	0.688	0.593	
DenseNet121	0.777	0.837	0.780 - 0.894	0.788	0.762	0.804	0.744	Train
MobileNet_v2	0.721	0.763	0.693 - 0.833	0.667	0.787	0.795	0.656	
CrossFormer	0.598	0.556	0.469 - 0.643	0.798	0.350	0.603	0.583	
ResNet50	0.733	0.775	0.638 - 0.912	0.692	0.789	0.818	0.652	
ResNet101	0.733	0.785	0.649 - 0.922	0.654	0.842	0.850	0.640	
DenseNet121	0.800	0.824	0.700 - 0.948	0.885	0.684	0.793	0.812	Internal validation
MobileNet_v2	0.756	0.704	0.539 - 0.870	0.962	0.474	0.714	0.900	
CrossFormer	0.600	0.516	0.335 - 0.698	0.692	0.474	0.643	0.529	
ResNet50	0.704	0.731	0.591 - 0.871	0.652	0.742	0.652	0.742	
ResNet101	0.722	0.760	0.631 - 0.890	0.739	0.710	0.654	0.786	
DenseNet121	0.759	0.840	0.736 - 0.944	0.870	0.677	0.667	0.875	Test 1
MobileNet_v2	0.815	0.820	0.702 - 0.939	0.826	0.806	0.760	0.862	
CrossFormer	0.630	0.599	0.445 - 0.753	0.609	0.645	0.560	0.690	
ResNet50	0.333	0.595	0.432 - 0.758	1.000	0.238	0.158	1.000	
ResNet101	0.740	0.697	0.498 - 0.897	0.667	0.750	0.276	0.940	
DenseNet121	0.792	0.794	0.658 - 0.930	0.750	0.798	0.346	0.957	Test 2
MobileNet_v2	0.750	0.842	0.745 - 0.940	0.833	0.738	0.312	0.969	
CrossFormer	0.469	0.587	0.437 - 0.738	0.833	0.417	0.169	0.946	

AUC, Area under curve; CI, Confidence interval; PPV, Positive predictive value; NPV, Negative predictive value; KNN, K-nearest neighbors; LR, Logistic regression; NB, Naïve Bayes; RF, Random forest.

Table S4: Performances of five DL methods in the 2D T2-FLAIR unimodal model.

Model	Accuracy	AUC	95% CI	Sensitivity	Specificity	PPV	NPV	Cohort
ResNet50	0.659	0.698	0.622 - 0.774	0.758	0.537	0.670	0.642	
ResNet101	0.693	0.732	0.659 - 0.805	0.596	0.812	0.797	0.619	
DenseNet121	0.637	0.690	0.614 - 0.767	0.485	0.825	0.774	0.564	Train
MobileNet_v2	0.626	0.662	0.583 - 0.742	0.525	0.750	0.722	0.561	
CrossFormer	0.553	0.442	0.358 - 0.526	1.000	0.000	0.553	0.000	
ResNet50	0.733	0.834	0.719 - 0.949	0.538	1.000	1.000	0.613	
ResNet101	0.733	0.822	0.701 - 0.943	0.538	1.000	1.000	0.613	
DenseNet121	0.711	0.751	0.606 - 0.896	0.731	0.684	0.760	0.650	Internal validation
MobileNet_v2	0.689	0.751	0.605 - 0.897	0.500	0.947	0.929	0.581	
CrossFormer	0.600	0.581	0.411 - 0.751	0.500	0.737	0.722	0.519	
ResNet50	0.778	0.853	0.754 - 0.952	0.870	0.710	0.690	0.880	
ResNet101	0.796	0.829	0.718 - 0.940	0.696	0.871	0.800	0.794	
DenseNet121	0.759	0.777	0.647 - 0.908	0.826	0.710	0.679	0.846	Test 1
MobileNet_v2	0.759	0.759	0.626 - 0.892	0.522	0.935	0.857	0.725	
CrossFormer	0.519	0.418	0.263 - 0.573	0.435	0.581	0.435	0.581	
ResNet50	0.688	0.812	0.691 - 0.934	0.833	0.667	0.263	0.966	
ResNet101	0.635	0.832	0.739 - 0.926	1.000	0.583	0.255	1.000	
DenseNet121	0.625	0.757	0.635 - 0.879	0.917	0.583	0.239	0.980	Test 2
MobileNet_v2	0.583	0.744	0.619 - 0.869	0.917	0.536	0.220	0.978	
CrossFormer	0.625	0.617	0.435 - 0.799	0.667	0.619	0.200	0.929	

AUC, Area under curve; CI, Confidence interval; PPV, Positive predictive value; NPV, Negative predictive value; KNN, K-nearest neighbors; LR, Logistic regression; NB, Naïve Bayes; RF, Random forest.

Table S5: Performances of five DL methods in the 2.5D T1CE unimodal model.

Model	Accuracy	AUC	95% CI	Sensitivity	Specificity	PPV	NPV	Cohort
ResNet50	0.721	0.777	0.710 - 0.844	0.677	0.775	0.788	0.660	
ResNet101	0.687	0.760	0.690 - 0.830	0.606	0.787	0.779	0.618	
DenseNet121	0.844	0.921	0.884 - 0.958	0.798	0.900	0.908	0.783	Train
MobileNet_v2	0.749	0.828	0.769 - 0.887	0.697	0.812	0.821	0.684	
CrossFormer	0.620	0.574	0.488 - 0.661	0.798	0.400	0.622	0.615	
ResNet50	0.822	0.874	0.772 - 0.977	0.692	1.000	1.000	0.704	
ResNet101	0.733	0.773	0.634 - 0.912	0.731	0.737	0.792	0.667	
DenseNet121	0.822	0.822	0.698 - 0.946	0.731	0.947	0.950	0.720	Internal validation
MobileNet_v2	0.800	0.828	0.706 - 0.950	0.808	0.789	0.840	0.750	
CrossFormer	0.689	0.579	0.396 - 0.762	0.769	0.579	0.714	0.647	
ResNet50	0.778	0.805	0.688 - 0.922	0.783	0.774	0.720	0.828	
ResNet101	0.722	0.729	0.593 - 0.866	0.435	0.935	0.833	0.690	
DenseNet121	0.852	0.874	0.768 - 0.980	0.783	0.903	0.857	0.848	Test 1
MobileNet_v2	0.796	0.884	0.798 - 0.970	0.913	0.710	0.700	0.917	
CrossFormer	0.630	0.609	0.455 - 0.762	0.870	0.452	0.541	0.824	
ResNet50	0.812	0.778	0.606 - 0.949	0.750	0.821	0.375	0.958	
ResNet101	0.594	0.792	0.679 - 0.904	1.000	0.536	0.235	1.000	
DenseNet121	0.729	0.867	0.766 - 0.968	0.917	0.702	0.306	0.983	Test 2
MobileNet_v2	0.760	0.841	0.748 - 0.935	0.917	0.738	0.333	0.984	
CrossFormer	0.750	0.683	0.521 - 0.845	0.583	0.774	0.269	0.929	

AUC, Area under curve; CI, Confidence interval; PPV, Positive predictive value; NPV, Negative predictive value; KNN, K-nearest neighbors; LR, Logistic regression; NB, Naïve Bayes; RF, Random forest.

Table S6: Performances of five DL methods in the 2.5D T2-FLAIR unimodal model.

Model	Accuracy	AUC	95% CI	Sensitivity	Specificity	PPV	NPV	Cohort
ResNet50	0.676	0.707	0.631 - 0.784	0.677	0.675	0.720	0.628	
ResNet101	0.670	0.731	0.658 - 0.804	0.687	0.650	0.708	0.627	
DenseNet121	0.771	0.834	0.776 - 0.893	0.717	0.837	0.845	0.705	Train
MobileNet_v2	0.687	0.737	0.665 - 0.809	0.596	0.800	0.787	0.615	
CrossFormer	0.525	0.512	0.427 - 0.597	0.303	0.800	0.652	0.481	
ResNet50	0.778	0.751	0.598 - 0.904	0.923	0.579	0.750	0.846	
ResNet101	0.600	0.603	0.436 - 0.770	0.462	0.789	0.750	0.517	
DenseNet121	0.800	0.802	0.668 - 0.936	0.962	0.579	0.758	0.917	Internal validation
MobileNet_v2	0.822	0.854	0.739 - 0.969	0.846	0.789	0.846	0.789	
CrossFormer	0.600	0.526	0.345 - 0.708	0.692	0.474	0.643	0.529	
ResNet50	0.778	0.794	0.672 - 0.916	0.826	0.742	0.704	0.852	
ResNet101	0.722	0.721	0.584 - 0.858	0.609	0.806	0.700	0.735	
DenseNet121	0.889	0.938	0.875 - 1.000	0.870	0.903	0.870	0.903	Test 1
MobileNet_v2	0.667	0.764	0.639 - 0.889	1.000	0.419	0.561	1.000	
CrossFormer	0.741	0.698	0.554 - 0.843	0.565	0.871	0.765	0.730	
ResNet50	0.875	0.756	0.590 - 0.922	0.500	0.929	0.500	0.929	
ResNet101	0.771	0.811	0.662 - 0.959	0.833	0.762	0.333	0.970	
DenseNet121	0.677	0.874	0.784 - 0.964	1.000	0.631	0.279	1.000	Test 2
MobileNet_v2	0.719	0.717	0.545 - 0.890	0.833	0.702	0.286	0.967	
CrossFormer	0.229	0.428	0.247 - 0.608	1.000	0.119	0.140	1.000	

AUC, Area under curve; CI, Confidence interval; PPV, Positive predictive value; NPV, Negative predictive value; KNN, K-nearest neighbors; LR, Logistic regression; NB, Naïve Bayes; RF, Random forest.

Table S7: MRI acquisition parameters for T1CE and T2-FLAIR across three centers.

Parameter	Center 1	Center 2	Center 3 (TCIA)
**T1CE**			
TR (ms)	464.59 ± 151.05	556.00 ± 91.09	1184 ± 729
TE (ms)	10.01 ± 2.64	9.67 ± 0.72	8.21 ± 5.65
FOV (cm)	240×240	256×256	224×224
Slice thickness (mm)	4.65 ± 1.07	5.16 ± 1.74	3.23 ± 2.21
**T2-FLAIR**			
TR (ms)	7506.46 ± 2696.34	7243.04 ± 2768.98	8592 ± 1383
TE (ms)	111.64 ± 46.83	94.84 ± 42.75	124 ± 89
FOV (cm)	220×220	256×256	4.70 ± 1.46
Slice thickness (mm)	4.96 ± 0.38	5.64 ± 0.50	224×224

Center 1: Cancer Hospital, Chinese Academy of Medical Sciences

Center 2: The First Affiliated Hospital of Anhui Medical University

Center 3: TCIA (Brain-Mets-Lung-MRI-Path-Segs)

TR，Repetition Time; TE, Echo Time; FOV, Field of View.

Table S8: Comparison of DriverNet with previously published MRI-based molecular prediction studies in NSCLC brain metastases.

Study	Endpoint	N	Imaging Input	Model Type	External Validation	AUC (Train / Internal / External)
Chen et al., 2020 [17]	EGFR	110	MRI radiomics	Random Forest	No	0.912 /NR / NR
	ALK	110	MRI radiomics	Random Forest	No	0.915 / NR / NR
	KRAS	110	MRI radiomics	Random Forest	No	0.985 /NR / NR
Cao et al., 2022 [18]	EGFR	162	T1CE + T2W MRI	Radiomics	Yes	0.968 / 0.901 / 0.900
Haim et al., 2022 [20]	EGFR	59	T1CE MRI	Deep learning	No	NR / 0.91 / NR
Li et al., 2024 [21]	19Del EGFR	177	Multi-sequence MRI	Deep learning	Yes	NR / 0.996 /1.000
	21L858R EGFR	177	Multi-sequence MRI	Deep learning	Yes	NR / 0.971 /0.991
	WT EGFR	177	Multi-sequence MRI	Deep learning	Yes	NR / 1.000 /1.000
DriverNet (Current Study)	EGFR/ALK	374	Clinical + T1CE + T2-FLAIR MRI	2.5D CNN + Transformer Fusion	Two independent external cohorts	0.967 / 0.947 / 0.962 and 0.952

NR, not reported; MRI, magnetic resonance imaging; T1CE, contrast-enhanced T1-weighted imaging; T2W, T2-weighted imaging; WT, wild type; AUC, area under the receiver operating characteristic curve.

Table S9: Computational complexity of the evaluated deep-learning architectures and the final DriverNet framework.

Model	Parameters (M)	FLOPs (G)
ResNet50	23.512	4.132
ResNet101	42.504	7.864
DenseNet121	6.956	2.896
MobileNet_v2	2.226	0.326
CrossFormer	28.269	4.656
DriverNet (Current Study)	10.147	5.802

Parameters denote the number of trainable model parameters. FLOPs denote the approximate floating-point operations for a single forward pass using an input size of 224 × 224 × 3. DriverNet values were calculated from the implemented architecture.

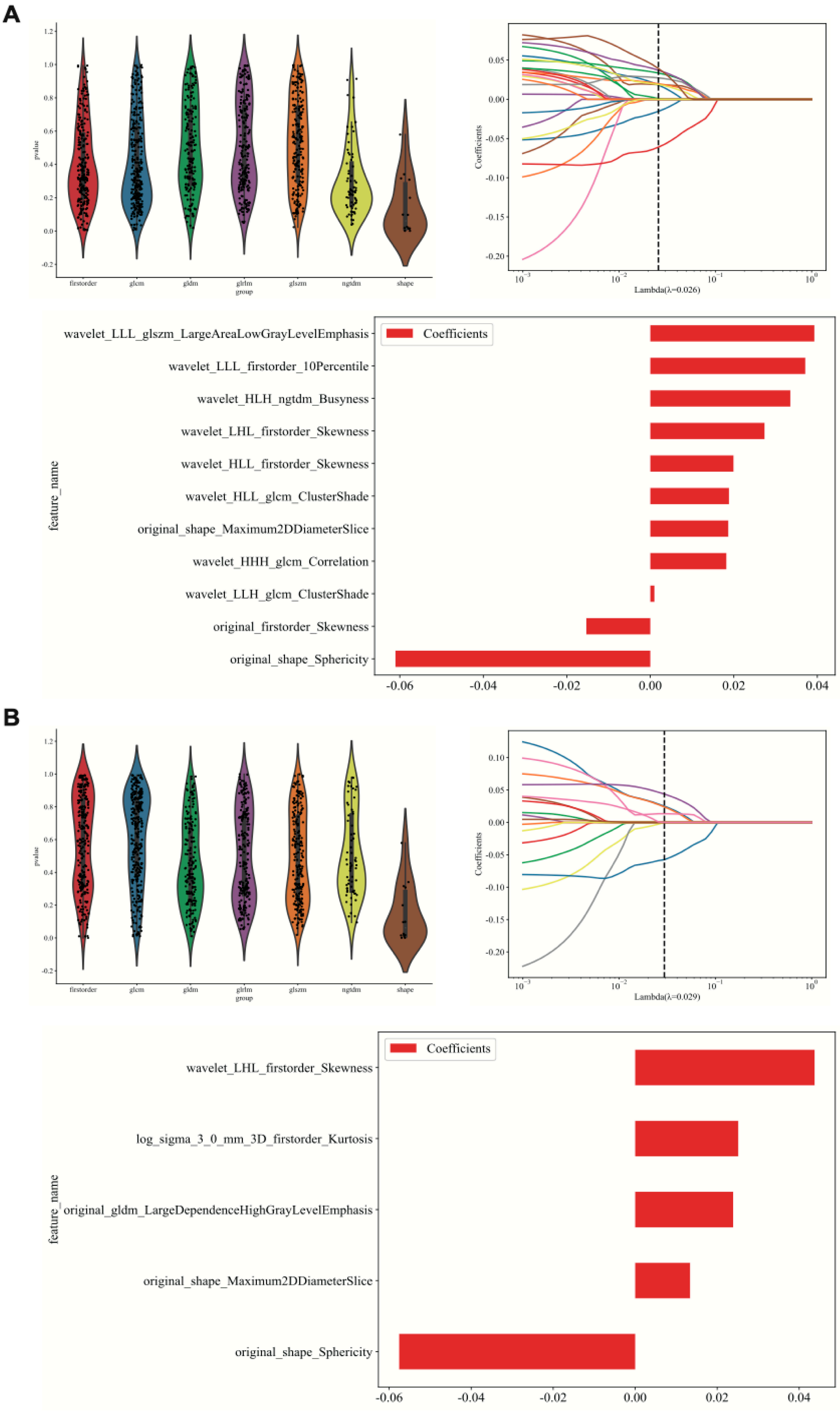
A
firstorder
glcm
gldm
glrlm
group
glszm
ngtdm
shape
pvalue
Coefficients
Lambda(λ=0.026)
Coefficients
wavelet_LLL_glszm_LargeAreaLowGrayLevelEmphasis
wavelet_LLL_firstorder_10Percentile
wavelet_HLH_ngtdm_Busyness
wavelet_LHL_firstorder_Skewness
wavelet_HLL_firstorder_Skewness
wavelet_HLL_glcm_ClusterShade
original_shape_Maximum2DDiameterSlice
wavelet_HHH_glcm_Correlation
wavelet_LLH_glcm_ClusterShade
original_firstorder_Skewness
original_shape_Sphericity
feature_name
B
firstorder
glcm
gldm
glrlm
group
glszm
ngtdm
shape
pvalue
Coefficients
Lambda(λ=0.029)
Coefficients
wavelet_LHL_firstorder_Skewness
log_sigma_3_0_mm_3D_firstorder_Kurtosis
original_gldm_LargeDependenceHighGrayLevelEmphasis
original_shape_Maximum2DDiameterSlice
original_shape_Sphericity
feature_name

Figure S1. Radiomics feature distribution and LASSO-based feature selection.

(A) T1CE radiomics. Left: violin plots show the normalized feature-value distributions across radiomics feature families (first-order, GLCM, GLDM, GLRLM, GLSZM, NGTDM, and shape). Top-right: LASSO coefficient profiles as a function of log(λ); the dashed vertical line denotes the optimal penalty (λ = 0.026). Bottom: non-zero features retained at the optimal λ with their corresponding coefficients.

(B) T2-FLAIR radiomics. Left: violin plots depict normalized feature-value distributions across the same feature families. Top-right: LASSO coefficient paths with the optimal penalty indicated by the dashed vertical line (λ = 0.029). Bottom: non-zero selected features and coefficients at the optimal λ.

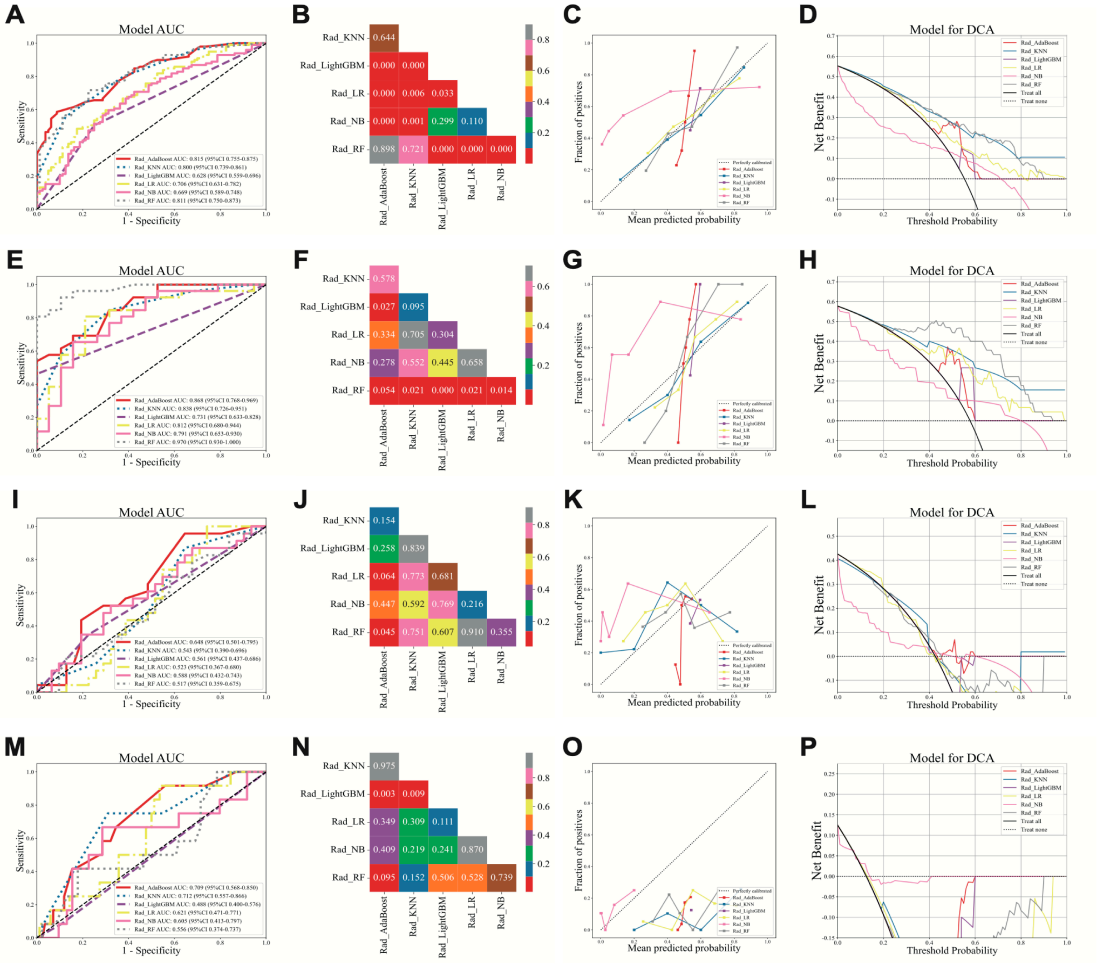

Figure S2. Performance comparison of six ML classifiers for the Rad T1CE model.

Radiomics features extracted from T1CE were used to build six classifiers—AdaBoost, k-nearest neighbors (KNN), LightGBM, logistic regression (LR), naïve Bayes (NB), and random forest (RF)—and to compare their performance across four cohorts. **(A–D) Training set; (E–H) internal validation set; (I–L) test set 1; and (M–P) test set 2.** Within each cohort, discrimination is assessed by receiver operating characteristic (ROC) curves and area under the curve (AUC) with 95% confidence intervals; pairwise AUC differences are summarized using DeLong test P values; calibration is evaluated by calibration curves (diagonal line indicates perfect calibration); and clinical utility is quantified by decision curve analysis (DCA) showing net benefit across threshold probabilities, with "treat-all" and "treat-none" reference strategies.

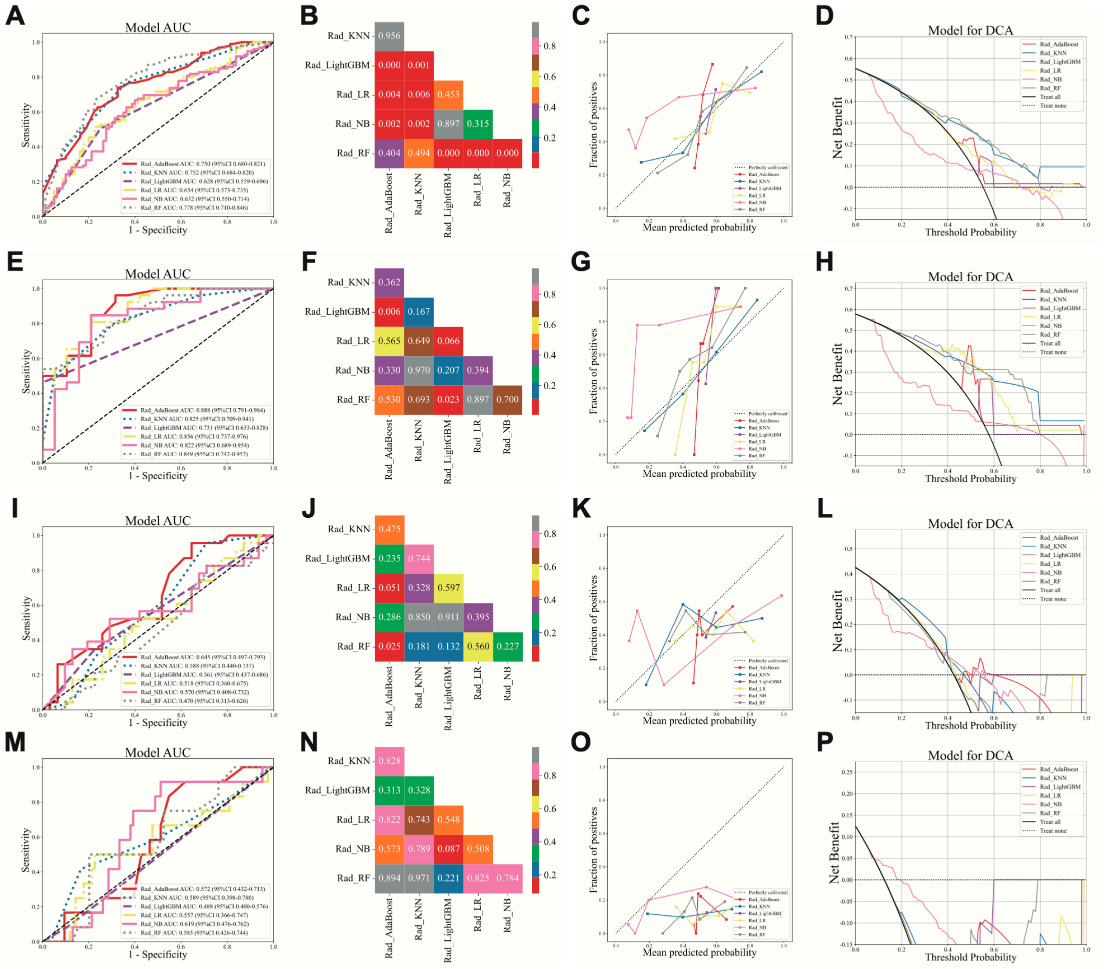

Figure S3. Performance comparison of six ML classifiers for the Rad T2-FLAIR model. Radiomics features extracted from T2-FLAIR were used to build six classifiers—AdaBoost, k-nearest neighbors (KNN), LightGBM, logistic regression (LR), naïve Bayes (NB), and random forest (RF)—and to compare their performance across four cohorts. **(A–D) Training set; (E–H) internal validation set; (I–L) test set 1; and (M–P) test set 2.** Within each cohort, discrimination is assessed by receiver operating characteristic (ROC) curves and area under the curve (AUC) with 95% confidence intervals; pairwise AUC differences are summarized using DeLong test P values; calibration is evaluated by calibration curves (diagonal line indicates perfect calibration); and clinical utility is quantified by decision curve analysis (DCA) showing net benefit across threshold probabilities, with "treat-all" and "treat-none" reference strategies.

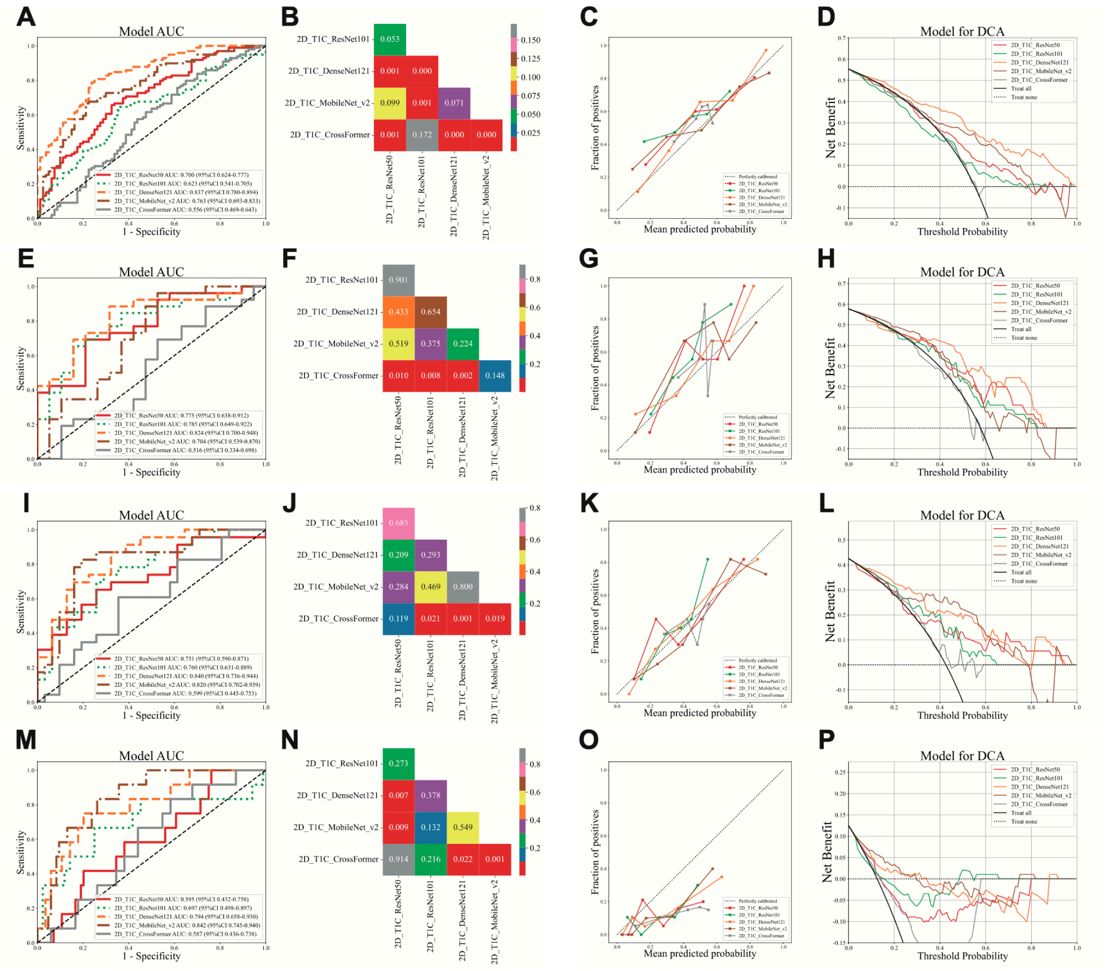

Figure S4. Performance comparison of five DL classifiers for the 2D T1CE model.

Five 2D DL classifiers: ResNet50, ResNet101, DenseNet121, MobileNet_v2, and CrossFormer, were trained using T1CE images and evaluated in four cohorts. **(A–D) Training set; (E–H) internal validation set; (I–L) test set 1; and (M–P) test set 2.** Within each cohort, discrimination is assessed by receiver operating characteristic (ROC) curves and area under the curve (AUC) with 95% confidence intervals; pairwise AUC differences are summarized using DeLong test P values; calibration is evaluated by calibration curves (diagonal line indicates perfect calibration); and clinical utility is quantified by decision curve analysis (DCA) showing net benefit across threshold probabilities, with “treat-all” and “treat-none” reference strategies.

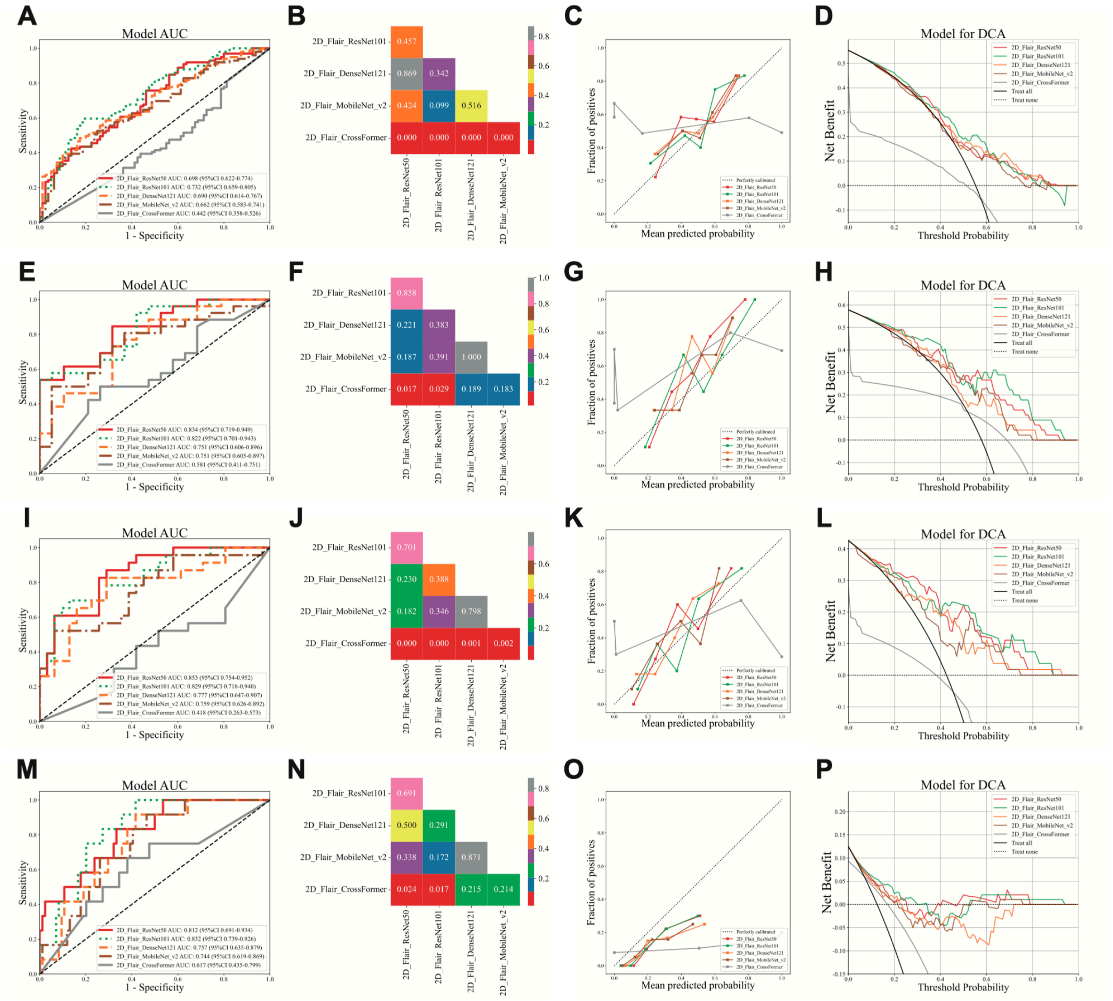

Figure S5. Performance comparison of five DL classifiers for the 2D T2-FLAIR model.

Five 2D DL classifiers: ResNet50, ResNet101, DenseNet121, MobileNet_v2, and CrossFormer, were trained using T2-FLAIR images and evaluated in four cohorts. **(A–D) Training set; (E–H) internal validation set; (I–L) test set 1; and (M–P) test set 2.** Within each cohort, discrimination is assessed by receiver operating characteristic (ROC) curves and area under the curve (AUC) with 95% confidence intervals; pairwise AUC differences are summarized using DeLong test P values; calibration is evaluated by calibration curves (diagonal line indicates perfect calibration); and clinical utility is quantified by decision curve analysis (DCA) showing net benefit across threshold probabilities, with “treat-all” and “treat-none” reference strategies.

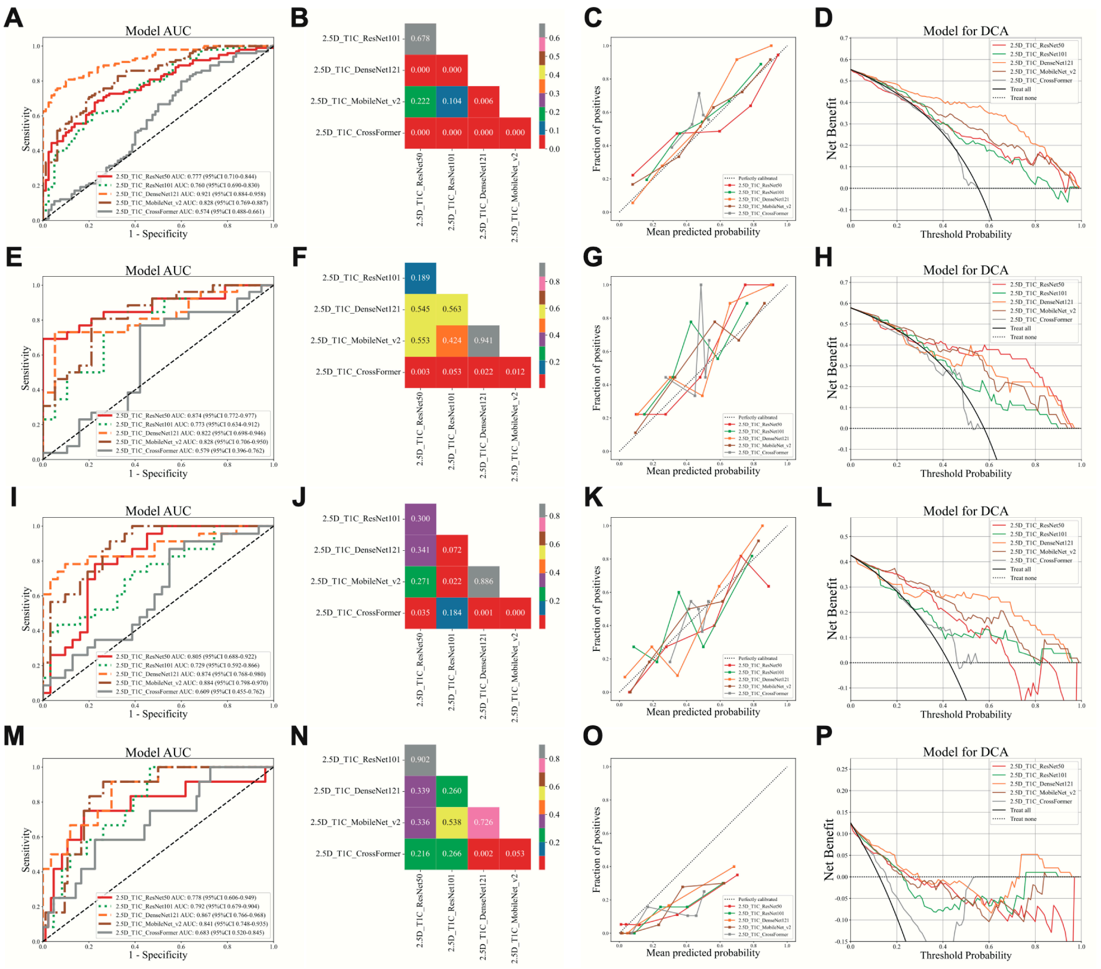

Figure S6. Performance comparison of five DL classifiers for the 2.5D T1CE model.

Five 2.5D DL classifiers: ResNet50, ResNet101, DenseNet121, MobileNet_v2, and CrossFormer, were trained using T1CE images and evaluated in four cohorts. **(A–D) Training set; (E–H) internal validation set; (I–L) test set 1; and (M–P) test set 2.** Within each cohort, discrimination is assessed by receiver operating characteristic (ROC) curves and area under the curve (AUC) with 95% confidence intervals; pairwise AUC differences are summarized using DeLong test P values; calibration is evaluated by calibration curves (diagonal line indicates perfect calibration); and clinical utility is quantified by decision curve analysis (DCA) showing net benefit across threshold probabilities, with “treat-all” and “treat-none” reference strategies.

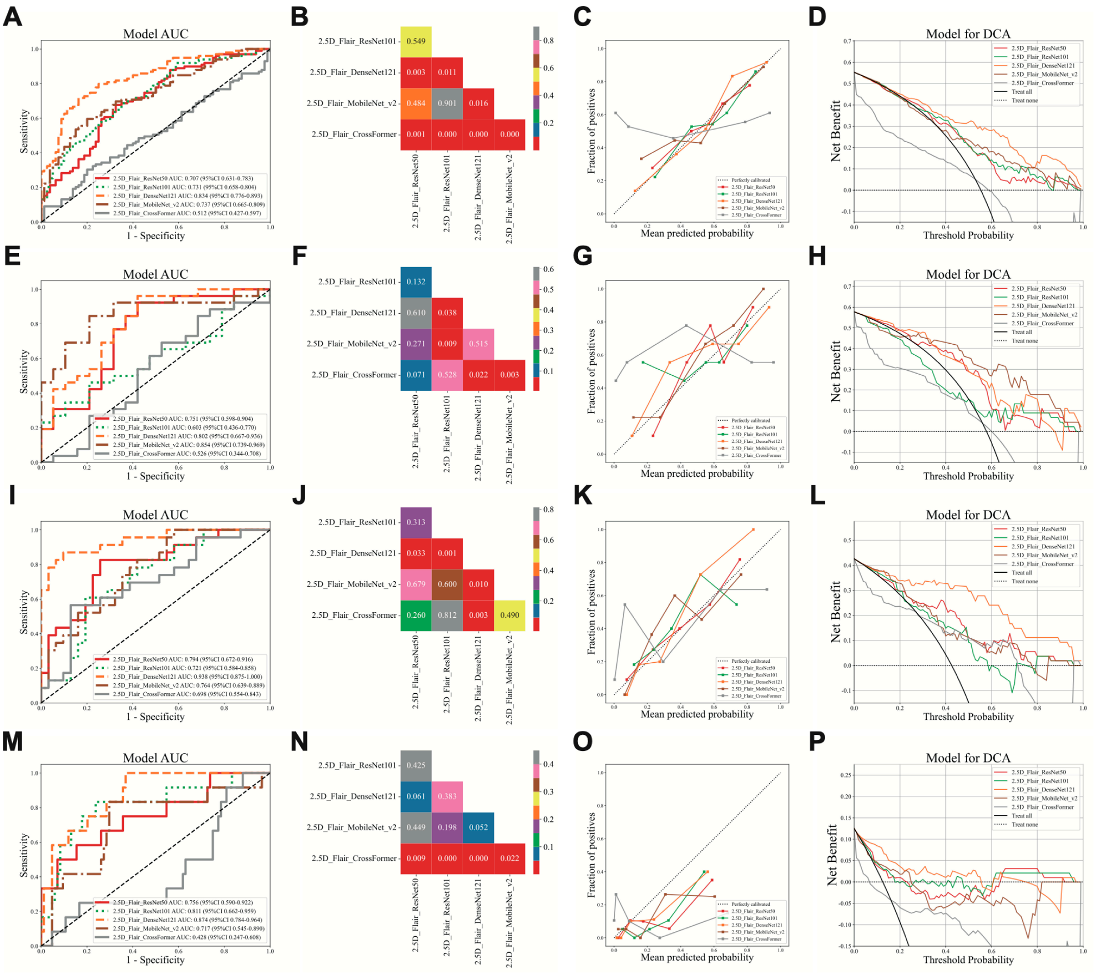

Figure S7. Performance comparison of five DL classifiers for the 2.5D T2-FLAIR model.

Five 2.5D DL classifiers: ResNet50, ResNet101, DenseNet121, MobileNet_v2, and CrossFormer, were trained using T2-FLAIR images and evaluated in four cohorts. **(A–D) Training set; (E–H) internal validation set; (I–L) test set 1; and (M–P) test set 2.** Within each cohort, discrimination is assessed by receiver operating characteristic (ROC) curves and area under the curve (AUC) with 95% confidence intervals; pairwise AUC differences are summarized using DeLong test P values; calibration is evaluated by calibration curves (diagonal line indicates perfect calibration); and clinical utility is quantified by decision curve analysis (DCA) showing net benefit across threshold probabilities, with “treat-all” and “treat-none” reference strategies.
